# Prevalence of mental health, mental well-being, and school connectedness in UK secondary school students in 2021/2022: Results from the South West - School Health Research Network pilot study

**DOI:** 10.1371/journal.pone.0355874

**Published:** 2026-08-28

**Authors:** Patricia N. Albers, Emily Widnall, Lorna Hatch, Georgina Hopkins, Catherine Sharp, Judi Kidger, Frank de Vocht, Eileen Kaner, Esther M. F. van Sluijs, Hannah Fairbrother, Russell Jago, Rona Campbell

**Affiliations:** 1 Population Health Sciences, University of Bristol, Bristol, United Kingdom; 2 Policy and International Health Directorate, a World Health Organization Collaborating Centre on Investment for Health and Well-Being, Public Health Wales, Wales, United Kingdom; 3 Population Health Sciences Institute, Newcastle University, Newcastle, United Kingdom; 4 MRC Epidemiology Unit, University of Cambridge, Cambridge, United Kingdom; 5 Health Sciences School, University of Sheffield, Sheffield, United Kingdom; Amrita Vishwa Vidyapeetham - Amritapuri Campus, INDIA

## Abstract

**Background:**

Young people are experiencing more mental health challenges, and connection to school can improve this. This study aimed to investigate adverse mental health, mental well-being, and school connectedness among secondary school students according to gender, academic year group, and socioeconomic status to identify areas for intervention.

**Methods:**

Self-report questionnaire data from 5193 Year 8 and 10 students (12−15 years) in the UK South West – School Health Research Network (SW-SHRN) were analysed. Weighted proportions and 95% Confidence Intervals (CIs) for all mental health and well-being (life satisfaction (Cantril Scale), depression (SMFQ), anxiety (GAD-7), mental well-being (SWEMWBS), loneliness (ONS measures for children)), and school connectedness outcomes were calculated, accounting for school clustering. Relationships between these and gender, academic year group, and socioeconomic status were explored using regression models.

**Results:**

A high proportion of respondents reported experiencing poor mental health, such as low life satisfaction (40%) and severe symptoms of depression (33%). Levels of school connectedness were also low; 47% of respondents reported a sense of belonging in school. Across all outcome measures, there was a gender gradient, boys experienced the most favourable and non-binary students the least favourable outcomes. Similarly, regression analyses confirmed gender as an important predictor, with especially stark differences between girls and boys in symptoms of depression [AOR 3.27 (CI: 2.84–3.76)] and anxiety [AOR 3.07 (CI: 2.62–3.61)]. Feeling safe in school and classmate support were associated with lower mental health outcomes whereas feeling a lot of schoolwork pressure predicted poorer outcomes, notably symptoms of depression [AOR 4.53 (CI: 3.40–6.08)] and anxiety [AOR 5.34 (CI: 3.79–7.66)].

**Conclusion:**

A high proportion of students reported poor mental health, mental well-being, and low school connectedness, with notable differences by gender. Our data suggest an urgent need for action and for schools to receive specific support to reverse deteriorating student mental health.

## Background

Mental health challenges among adolescents are increasing [1] and were exacerbated by the Covid-19 pandemic [2]. In England in 2017, it was estimated that one in nine children and young people experienced mental health challenges [3], which increased to one in six during the 2020 Covid-19 pandemic [4]. After this jump between 2017 and 2020, the rates remained relatively stable between 2020 and 2023, with about one in five children and young people having a probable mental disorder in 2023 [5].

Adolescence is a critical development period including a number of physical and emotional changes, heightened response to stressors [6], and marked increases in social sensitivity and the importance of seeking relationships outside of the family [7]. Half of all mental health disorders start by age 14 [8]. Unhealthy behaviours and risk-taking behaviours, including smoking, drinking, and drug use, often begin during adolescence and are closely related to a number of public health challenges [9–11].

Girls have consistently been found to suffer from greater levels of mental health problems in comparison to boys across a range of outcomes [12–15]. Population surveys of adolescents reveal that adolescent girls are more prone to experience internalising disorders such as anxiety or depression, whereas boys are more likely to experience externalising disorders or behaviours, such as aggression or conduct disorders [12,14,16,17]. Further, there is strong evidence that adolescents from lower socioeconomic status families, or with lower parental education levels have more mental health challenges compared to their peers [14,18–21].

School environment often refers to the relationships among members of the school community (such as students, teachers, support staff etc), these relationships are determined by structural, personal, and functional factors of the educational institution, which are unique to any particular school [22]. However, school environment can also refer to a schools’ organisation or management, teaching, pastoral care, discipline and/or physical environment [23].

The school environment, as well as how connected young people feel to their school and peers, is an integral component of adolescent mental health [24,25], both in terms of depressive symptoms and experiences of bullying. In a study looking at the mediating effect of school environment on adolescent mental health, results showed that supportive relationships accounted for 51% of the mediating effect of school environment on depressive symptoms [26]. Supportive relationships (57%), participation in school events (22%), and belonging in school (18%) were the subcomponents accounting for the mediating effect of school environment on experiences of bullying [26].

School connectedness is frequently defined as feeling cared for by staff and peers in an academic sense and individually, feeling accepted, respected, included, and supported within the school [27–29]. Research has demonstrated strong negative associations between low school connectedness and concurrent and future self-report symptoms of depression and anxiety [28–30]. School connectedness has also been found to predict depressive symptoms one year later in girls and boys as well as predict anxiety symptoms one year later in girls [30]. Additionally, according to a UK study using Health Behaviour in School-aged Children (HBSC) survey data, school students who reported a low sense of belonging to both school and the community had, 6.7 times and 2.8 times respectively, higher odds of reporting self-harm than those with a greater sense of belonging [31].

Linked to the school environment and connectedness is the experience of schoolwork pressure, characterised as a disparity between what a school demands and student’s personal capital or strengths to meet these [32]. Schoolwork contributes to adolescent stress impacting adolescent mental health and mental well-being and has been postulated as contributing to the decline in adolescent mental well-being [33].

Given the evidence surrounding the role of the school environment and school connectedness in determining adolescent mental health, there has been an increase in the use of whole-school mental health interventions, which target the entire school environment [34], as well as approaches that focus on general health promotion in schools. A 2019 meta-analysis which sought to determine the effectiveness of interventions adopting a whole-school approach to enhance young people’s social and emotional development found small, but significant, improvements in participants’ social and emotional adjustment, behavioural adjustment, and internalising symptoms [35]. The Health Promoting Schools (HPS) approach is a holistic method which moves beyond individual level behaviour change (centred around knowledge, attitudes, and behaviours) and instead focusses on the social, organisational, and/or environmental elements of the school [36]. HPS adopt a whole-school approach, where a comprehensive health education curriculum is reinforced by the school’s environment and values [37]. HPS also emphasises organisational change, by strengthening the physical and social environments, such as, interpersonal relationships, school management, policy structures, and teaching and learning conditions [37].

Students who feel more connected to their school are more likely to seek help or support [38]. Schools play a central role in help-seeking, with previous explorations of pathways into mental health services revealing approximately 60% of young people initially accessed mental health services via educational settings [39]. Schools offer a convenient and accessible way to reach young people meaning they can play a vital role in detecting health challenges and provide young people with support [40,41].

The aim of this paper was to explore the prevalence of mental health difficulties and levels of school connectedness among secondary school students, by gender, academic year group, and socioeconomic status, in South West England. A secondary aim was to explore the relationship between mental health and mental well-being outcomes with school connectedness, gender, academic year group, and socioeconomic status.

## Methods

### Study design

This paper reports data from the student health and well-being survey completed as part of the pilot study of the School Health Research Network (SHRN) based in South West England [42]. Full details on the design of this pilot study can be found in our protocol paper [42].

The overarching aim of this pilot was to establish a SHRN to improve the health and well-being of the students. SHRNs are a policy-practice-research partnership that aim to provide high quality health and well-being data for stakeholders to drive health improvement [43]. A co-operative approach between stakeholders within the education and health systems, namely local authorities, public health researchers, public health practitioners, school educators, young people, and parents was pursued during the development of the South West – School Health Research Network (SW-SHRN) [42].

Secondary schools, from seven local authorities, were recruited either via the local authority or by being approached by the study team directly. As part of the pilot we aimed to recruit 20 schools. Student recruitment took place between 02/03/2021 and 27/06/2022. All students in Years 8 (age 12–13) and 10 (age 14–15) in the participating schools were invited to complete a health and well-being survey. The self-report survey was completed electronically in a 45-minute school lesson. Self-report methods are frequently used in adolescent health surveys including the Health Behaviour in School-aged Children (HBSC) study and the wider School Health Research Network (SHRN) infrastructure. Confidential self-completed surveys have been shown to improve disclosure of sensitive topics among adolescents, such as mental health [44]. All survey questions were categorical with no open-ended questions, and all questions included an “I do not want to answer” option. The survey was refined with input from a Young Persons’ Advisory Group to ensure clarity, understandability, and age-appropriate language. The survey also included simplified explanations for commonly misunderstood terms. Further, the survey was administered during school time with researchers present, and participants were encouraged to seek clarification from researchers or staff when required. Data were collected via an electronic data capture tool; REDCap (Research Electronic Data Capture) [45,46] and analysed using R [47].

### Inclusion and exclusion criteria of schools and students

Only secondary schools within the fifteen local authorities in South West England were invited to join the network. Special schools and pupil referral units were not included in this pilot study, as these schools are not part of the majority school provision in England [48], and are better suited for inclusion once the SW-SHRN model has been created and tested within mainstream education settings.

Students were included if they were currently in Year 8 or Year 10, they had not been opted out of the survey by a parent or guardian, and they provided assent to participate. Students who fulfilled all of these criteria but then did not answer any questions or selected “I do not want to answer” to all questions within the survey, were excluded.

We included students in Years 8 and 10 in order to align with the sampling strategy used within the wider School Health Research Network (SHRN) model. This approach captures two distinct developmental and educational stages (Key Stage 3 and 4, in the English educational system) during secondary education: early adolescence (Year 8; aged 12–13 years) and mid-adolescence (Year 10; aged 14–15 years).

#### School characteristics.

In total, 18 schools completed the survey across the two academic years (2020/2021 and 2021/2022), with six schools taking part during the 2020/2021 academic year. Students in two of these six schools completed the survey remotely during home learning as a result of the Covid-19 pandemic restrictions and school closures. All remaining participating schools completed the survey after restrictions had eased and students had returned to in-person schooling, although schools continued to be influenced by the aftermath of the COVID-19 pandemic. In the two schools which completed the survey remotely they used their existing methods for maintaining contact and sharing learning materials with students, for instance online platforms and emails. However, in the broader context of the pandemic, schools varied in the methods and extent of contact with students during school closures.

Response rates ranged from 16% to 97%, with an overall response rate of 74%. The two schools that completed the survey whilst at home had the lowest response rates. Most of the schools are located in less deprived areas with five each in Index of Multiple Deprivation (IMD) quintiles one and two, and only two situated in each of the two most deprived IMD quintiles (quintiles four and five).

One school contributed two separate student cohorts as they completed the survey in both academic years. This was because the response rate was low (16%) in the first year because of the students being locked down at home during the Covid-19 pandemic.

Descriptive comparisons show variation between schools in school connectedness and mental health and well-being outcomes (S1 Table); however, the aim of this study was not to explore school-level determinants or contextual effects. School-level clustering was accounted for statistically in the prevalence analyses, but our analyses remained focused on individual-level associations.

### Measures

Our survey questions were based on those included in the HBSC survey, Welsh SHRN, and the Office for National Statistics [49–53]. The survey included validated adolescent health and well-being instruments widely used in school-based population health research. Only validated measures were used in the analyses and reported in this paper. Further details on all included measures, and how they were used in this paper, can be found in S2 Text. The demographic measures included were gender (girl, boy, or “neither word describes me”; hereafter referred to as ‘nonbinary’), age (based on students’ date of birth), socioeconomic status (measured by the Index of Multiple Deprivation [IMD] [54], derived from students’ household postcode provided by the school), ethnicity, disability, and family structure. For the prevalence estimates, IMD was treated as an ordinal variable ranging from 1 to 5 with 1 being the most deprived 20% of areas and 5 being the least deprived 20% of areas.

#### Mental health and mental well-being measures.

Our analyses included six measures of mental health, two measured loneliness, and the remaining covered life satisfaction, symptoms of depression, anxiety, and mental well-being. For loneliness, one comprised of a three part scale, while the other was a direct measure [53]. Anxiety was measured using the 7-item Generalised Anxiety Disorder – 7 (GAD-7) scale [55]. Symptoms of depression were measured using the 13-item Short Mood and Feelings Questionnaire (SMFQ) [56]. Mental well-being was measured using the 7-item Short Warwick-Edinburgh Mental Well-being Scale (SWEMWBS) [57], which is centred around functioning rather than emotions. Life satisfaction was measured using the Cantril Scale [58], on which respondents rate their overall life satisfaction on a scale of 0–10. Detailed explanation of the measures and their categorisation can be found in S2 Text.

#### School connectedness measures.

Feelings about school, schoolwork pressure, belonging in school, and feeling safe in school were all measured using single questions from the HBSC survey [49,50]. Classmate and teacher support were measured using three and four questions respectively also from the HBSC survey [49,50] to form two composite scales. Peer support was measured using the peer support subset of questions from the Multidimensional Scale of Perceived Social Support (MSPSS) [59]. Detailed explanation of the measures and their categorisation can be found in S2 Text.

### Statistical methods/analyses

Using the ‘survey’ package in R school level clustering and response rate weighting was accounted for in all the prevalence analyses [60]. Response rate weighting was based on school response rate such that schools with lower response rates were up weighted so responses were not underrepresented in the overall sample. Class level clustering could not be accounted for because of lack of appropriate data. All prevalence data were analysed using the ‘survey’ package in R (Version 4.4.1) [60]. Sample characteristics were reported, and all outcome data (mental health, mental well-being, and school connectedness) were analysed descriptively by gender, academic year group, and socioeconomic status. For most measures, we provide the proportion and associated 95% Confidence Interval (CI). For continuous variables the mean and standard deviation are provided. Pearson’s Chi-squared tests were used to explore associations between the outcome variables and the sub-group variables. Under the ‘survey’ package this Chi-squared statistic is adjusted and compared to the distribution under simple random sampling [60]. The p-value is calculated using the Satterthwaite approximation [60].

For all scale-based questions (symptoms of depression, anxiety, and mental well-being), a 20% missing data threshold was used, meaning if a student did not respond to 20% or more of the scale’s items, their total score was not calculated and their response was counted as missing. However, if a student missed less than 20% of the scale items, their average response to the answered items was imputed in the missing item’s place, resulting in a complete scale score. For the loneliness, classmate support, teacher support, and peer support scales if a student did not answer one of the scale questions, their final score was not calculated and their response was counted as missing.

#### Regression analyses.

Regression analyses were conducted for mental health and mental well-being outcomes [life satisfaction, depression, anxiety, mental well-being, loneliness (no one to talk to, feeling left out, feeling alone, direct question)] and gender, academic year group, socioeconomic status, and school connectedness predictors (feelings about school, schoolwork pressure, belonging in school, feeling safe in school, classmate support, teacher support, peer support). Students who identified as nonbinary were excluded from the analyses because of low numbers. All mental health and mental well-being outcomes were categorical, and were modelled using Ordinal Logistic Regression (OLR). Prior to running the OLR models all assumptions were checked. Where the proportional odds assumption was violated the most balanced binary form of the variable was used. Details of these checks and resultant binary variables are provided in S2 Text. For binary outcomes, logistic regression models were used.

Predictors included in each model were: gender, academic year group, socioeconomic status, feeling about school, schoolwork pressure, belonging in school, feeling safe in school, classmate support, teacher support, and peer support. Academic year group, feelings about school, and schoolwork pressure were treated as categorical variables, while socioeconomic status, classmate support, teacher support, and peer support were included as continuous variables.

Backwards elimination was used to remove non-significant (p-value >= 0.05) school connectedness variables from each model. Gender, academic year group, socioeconomic status were not removed regardless of their significance, allowing them to be controlled for in the final models. After each removal, the reduced model was compared, using ANOVA, with the previous model to assess whether the excluded variable made a significant contribution to model fit.

All regression analyses were conducted in R (Version 4.4.1) [47]. Complete case analysis was used, meaning cases with missing data on any variable included in the regression were removed from the analyses.

### Ethics

Ethical approval for this study was obtained through the University of Bristol, Faculty of Health Science Research Ethics committee (Ref. 110922). After schools agreed to participate, they electronically signed three agreement forms, which outlined the expectations for participating schools, a data sharing agreement, and a report usage agreement. Parental/guardian consent was obtained prior to the survey, through the use of an opt-out consent procedure. Parents/ guardians were sent an information sheet about the study, and a withdrawal form to return to the research team, or their child’s school, if they did not want their child to take part. On the day of data collection, researchers explained to students, who had not been opted-out by their parents/ guardians, the voluntary and confidential nature of the survey. Students were provided with an information sheet and electronic written informed assent was obtained from students prior to them completing the survey.

## Results

### Sample characteristics

Table 1 provides the sample demographics and gender stratification. In total 6868 student names were provided by the schools, of which 5193 (76%) completed the survey. Over 80% (4326) of respondents provided complete responses. Missing data across all the different variables were relatively low, < 10% for most, with some variables (family structure, classmate, teacher, and peer support) having slightly higher missing rates (>/ = 10%), but all were <17%. Frequencies and proportions across key measures for students with and without data on the student, teacher, and peer support measures were explored and presented in S3 Table. It is evident that the distribution across these variables was comparable between those with and without data. However, there is some indication that participants with missing data for the student support measure had slightly poorer outcomes, suggesting missingness may not have been completely random. Therefore, some bias in the effect estimates is possible. Further, students with missing data across either of the three variables were more often from lower socioeconomic backgrounds.

**Table 1:** Student sample demographics.

Demographics	n (%)	Boys n (%)	Girls n (%)	Nonbinary n (%)	Missing n (%)
**Student sample:**	5193	2625 (51)	2322 (46)	160 (3)	
*Completed full survey*	4326 (83)	2166 (51)	1954 (46)	131 (3)	
**Age:**					22 (0.4)
*12 years*	1316 (26)	662 (25)	590 (26)	39 (24)	
*13 years*	1417 (27)	715 (27)	641 (28)	38 (24)	
*14 years*	1170 (23)	586 (22)	513 (22)	46 (29)	
15 years*	1268 (25)	651 (25)	567 (25)	37 (23)	
**Academic year group:**					4 (<0.1)
*Year 8*	2744 (53)	1383 (53)	1236 (53)	77 (48)	
*Year 10*	2445 (47)	1241 (47)	1083 (47)	83 (52)	
**Ethnicity**					77 (1.5)
*White*	4404 (86)	2206 (85)	2004 (87)	127 (83.01)	
*Mixed/Multiple ethnic groups*	236 (5)	135 (5)	91 (4)	5 (3)	
*Asian/Asian British*	276 (5)	147 (6)	117 (5)	10 (7)	
*Black/African/Caribbean/Black British*	123 (2)	64 (2)	53 (2)	5 (3)	
*Arab*	32 (1)	17 (1)	9 (<1)	5 (3)	
*Other*	45 (1)	17 (1)	26 (1)	1 (1)	
**Socioeconomic status**					42 (0.8)
*1 (Least deprived)*	1593 (31)	800 (31)	720 (31)	43 (27)	
*2*	1394 (27)	694 (27)	629 (27)	44 (28)	
*3*	959 (19)	487 (19)	431 (19)	29 (18)	
*4*	632 (12)	328 (13)	274 (12)	19 (12)	
*5 (Most deprived)*	573 (11)	293 (11)	249 (11)	25 (16)	
**Having a disability**	570 (11)	238 (9)	277 (12)	38 (27)	189 (3.6)
*Disability impacts school attendance*	234 (45)	84 (38)	129 (50)	16 (50)	
**Family structure:**					
Number of adults living in household					878 (16.9
*1*	569 (13)	280 (13)	252 (13)	21 (16)	
*2*	2913 (68)	1480 (68)	1305 (67)	88 (66)	
*3 +*	833 (19)	404 (19)	391 (20)	25 (19)	
Number of siblings					873 (16.8
*Only child*	942 (22)	480 (22)	407 (21)	37 (28)	
*1 or 2*	2805 (65)	1391 (64)	1294 (66)	78 (58)	
*3 or 4*	496 (12)	250 (12)	225 (12)	15 (11)	
*5 +*	77 (2)	45 (2)	27 (1)	4 (3)	

*One student was 16 years old, they were recoded as being 15 years old

There were slightly more Year 8 respondents than Year 10 respondents. The majority (86%) reported their ethnic group as ‘white’. The largest proportion (31%) of students resided in the least deprived areas, while the smallest proportion (11%) resided in the most deprived areas. Just over 10% reported having a long-term health problem or disability, and nearly half (45%) of those said it impacted their school attendance or participation. The majority (68%) of respondents lived in a household with two adults and the majority (65%) had one or two siblings. 3% of respondents reported their gender as nonbinary.

### Mental health and mental well-being

Table 2 shows the unweighted and weighted sample proportions for all the mental health, mental well-being, and school connectedness variables. 40% of respondents reported low life satisfaction, and 33% reported severe symptoms of depression (score >=12 out of 26), with a further 16% reporting probable depression (Table 2). 17% of respondents reported severe anxiety, and a further 17% had moderate levels of anxiety. For mental well-being, 21% were ranked as having poor mental well-being (score <=17), with a further 18% having low mental well-being (score >=18 & <=20). 13% of respondents reported feeling lonely often and an additional 21% reported feeling lonely some of the time. We found a high correlation between the students’ responses to this direct question and the loneliness scale (Adjusted R-Squared: 0.68, p < 0.001).

**Table 2:** Unweighted and weighted sample proportions for all mental health, mental well-being, and school connectedness measures.

	Missing n (%)	Unweighted proportion (%)	Weighted proportion (%)	95% Confidence Interval
**Mental health and mental well-being**				
**Life satisfaction**	137 (3.2)			
*High*		17	17	15–18
*Average*		43	44	41–46
*Low*		40	40	37–43
**Depression**	154 (3.0)			
*No depression*		51	51	47–54
*Probable depression*		16	16	15–17
*Severe depression*		33	33	31–36
**Anxiety**	163 (3.1)			
*None to low*		41	41	37–44
*Mild*		24	25	23–26
*Moderate*		17	17	16–19
*Severe*		17	17	16–19
**Mental well-being**	146 (2.8)			
*High*		16	16	14–19
*Average*		45	45	44–47
*Low*		18	18	16–19
*Poor*		21	21	18–23
**Loneliness (No one to talk to)**	166(3.2)			
*Hardly*		45	45	43–47
*Some*		35	35	34–37
*Often*		20	20	18–22
**Loneliness (Feeling left out)**	152 (2.9)			
*Hardly*		41	41	39–43
*Some*		39	39	38–40
*Often*		20	20	18–22
**Loneliness (Feeling alone)**	168 (3.2)			
*Hardly*		48	48	45–50
*Some*		32	32	31–33
*Often*		20	20	18–22
**Loneliness (Scale of the above three)**	219 (4.2)			
*Mean (SD)*		5.3 (2.0)	5.3 (2.0)	–
**Loneliness (Direct question)**	151 (2.9)			
*Never*		17	17	16–18
*Hardly ever*		27	27	25–29
*Occasionally*		22	22	21–24
*Sometimes*		20	21	19–22
*Often*		13	13	11–14
**School connectedness**				
**Feelings about school**	352 (6.8)			
*Like it a lot*		11	11	7–15
*Like it a bit*		42	42	40–45
*Don’t like it very much*		28	28	25–30
*Don’t like it at all*		19	19	17–22
**Schoolwork pressure**	340 (6.5)			
*Not at all*		12	12	9–14
*A little*		31	31	29–33
*Some*		26	26	24–27
*A lot*		32	32	29–35
**I feel like I belong in this school**	496 (9.6)			
*Agree*		47	47	42–53
*Disagree*		53	53	47–58
**I feel safe in this school**	508 (9.8)			
*Agree*		52	52	45–59
*Disagree*		48	48	41–55
**Classmate support**	551 (10.6)			
*Very high*		14	14	10–18
*High*		21	21	19–23
*Low*		30	30	28–32
*Very low*		36	35	30–40
**Teacher support**	575 (11.1)			
*Very high*		20	20	17–23
*High*		26	26	24–27
*Low*		22	22	20–23
*Very low*		32	33	29–36
**Peer support**	699 (13.5)			
*High*		46	46	43–49
*Moderate*		27	27	25–28
*Low*		27	27	24–30

### School connectedness

Nearly half (47%) of respondents reported not liking school very much or at all. Around a third (32%) of respondents felt a lot of schoolwork pressure, and a further 26% felt some schoolwork pressure. Nearly half (47%) reported a sense of belonging in school and only just over half (52%) reported feeling safe in school. Nearly half (46%) of respondents reported feeling supported by their teachers, and similarly nearly half (46%) reported high levels of peer support. While only 35% reported high or very high levels of classmate support.

### Mental health, mental well-being, and school connectedness by gender

Table 3 shows the weighted proportions for all the mental health, mental well-being, and school connectedness measures by gender (girls, boys, or nonbinary). For all the mental health and mental well-being outcomes there was a difference between the three gender groups. This difference between the gender groups was most notable for symptoms of severe depression, where 73% of nonbinary respondents reported symptoms of severe depression, compared to 47% of girls, and only 17% of boys. Similarly, for poor mental well-being, nonbinary respondents (50%) and girls (28%) had substantially higher prevalence than boys (12%).

**Table 3:** Weighted proportions for all mental health, mental well-being, and school connectedness measures by gender.

	Boys		Girls		Nonbinary		
	%	95% CI	%	95% CI	%	95% CI	P-value
**Mental health and mental well-being**							
**Life satisfaction**							<0.001
*High*	20	18–22	14	12–16	9	6–13	
*Average*	51	49–53	38	35–41	20	14–26	
Low	30	27–32	48	44–52	71	63–78	
**Depression**							<0.001
*No depression*	67	63–71	35	32–38	16	10–22	
*Probable depression*	15	13–17	18	16–19	12	8–16	
*Severe depression*	18	15–20	48	45–51	73	65–80	
**Anxiety**							<0.001
*None to low*	56	52–60	26	22–29	15	9–20	
*Mild*	25	22–27	25	23–27	17	11–23	
*Moderate*	12	10–14	22	21–24	30	22–37	
*Severe*	8	6–9	27	24–30	39	33–45	
**Mental well-being**							<0.001
*High*	23	19–26	10	8–12	7	3–10	
*Average*	51	49–52	41	39–43	26	19–32	
*Low*	15	13–17	21	19–23	18	12–24	
*Poor*	12	10–14	28	25–31	50	42–58	
**Loneliness (No one to talk to)**							<0.001
*Hardly*	57	55–60	33	30–36	18	12–23	
*Some*	30	29–32	40	37–43	41	33–50	
*Often*	12	11–13	27	24–30	41	34–48	
**Loneliness (Feeling left out)**							<0.001
*Hardly*	53	50–56	29	27–31	20	13–28	
*Some*	35	33–37	44	42–46	33	27–40	
*Often*	12	11–14	27	24–29	46	36–56	
**Loneliness (Feeling alone)**							<0.001
*Hardly*	61	58–64	36	34–39	17	13–21	
*Some*	27	25–30	37	34–39	32	26–38	
*Often*	12	10–13	27	25–30	51	43–58	
**Loneliness (Scale of above three)**							
*Mean (SD)*	4.64	(0.06)	5.82	(0.07)	6.84	(0.17)	
**Loneliness (Direct question)**							<0.001
*Never*	25	24–26	10	9–11	6	3–8	
*Hardly ever*	34	31–36	22	20–23	14	9–19	
*Occasionally*	20	18–22	26	24–27	14	8–20	
*Sometimes*	14	25–28	26	13–16	34	27–40	
*Often*	7	6–8	17	15–19	33	25–41	
**School connectedness**							
**Feelings about school**							<0.001
*Like it a lot*	13	8–17	9	6–12	6	2–9	
*Like it a bit*	47	45–50	38	35–41	27	17–38	
*Don’t like it very much*	26	23–29	29	26–32	32	25–38	
*Don’t like it at all*	14	11–16	24	21–27	35	27–44	
**Schoolwork pressure**							<0.001
*Not at all*	16	13–19	7	5–9	7	2–12	
*A little*	37	35–39	26	23–28	19	13–25	
*Some*	25	23–27	27	25–29	18	11–24	
*A lot*	22	19–25	40	37–44	57	50–64	
**I feel like I belong in this school**							<0.001
*Agree*	57	51–62	40	34–45	19	9–29	
*Disagree*	43	38–49	60	55–66	81	71–91	
**I feel safe in this school**							<0.001
*Agree*	59	52–66	47	40–53	24	9–38	
*Disagree*	41	34–48	53	47–60	76	62–91	
**Classmate support**							<0.001
*Very high*	19	14–24	10	7–12	6	3–9	
*High*	25	23–26	17	14–21	8	4–12	
*Low*	30	27–33	31	29–33	20	12–27	
*Very low*	27	22–31	42	36–47	67	57–76	
**Teacher support**							<0.001
*Very high*	23	20–26	17	14–20	13	7–20	
*High*	28	26–30	24	22–26	18	14–23	
*Low*	21	19–22	23	21–25	22	15–29	
*Very low*	28	25–31	37	32–41	46	37–55	
**Peer support**							0.005
*High*	44	40–48	49	46–52	46	35–57	
*Moderate*	29	27–31	24	22–26	25	17–32	
*Low*	27	24–30	28	24–31	29	22–36	

For all the school connectedness measures there was a difference between the three gender groups. Peer support was the only measure where the patterns were more alike for the different groups. Similar to the mental health and mental well-being outcomes, nonbinary respondents reported lower school connectedness than girls and boys. This was most striking for belonging in school and feeling safe in school, where 19% of nonbinary students reported that they felt they belonged in school, compared to 40% of girls and 57% of boys. Similarly, only 24% of nonbinary students reported feeling safe in school, compared to 47% of girls and 59% of boys.

### Mental health, mental well-being, and school connectedness by academic year group

Table 4 provides the weighted mental health, mental well-being, and school connectedness measures across Year 8 and Year 10 respondents. There was a difference between all measures except for two: loneliness (no one to talk to) and loneliness (feeling left out). Across the mental health and mental well-being measures, Year 10 respondents typically reported poorer outcomes than Year 8 respondents. The largest contrast was found in the proportion of Year 8 and Year 10 respondents who reported low levels of life satisfaction, 35% and 45%, respectively.

**Table 4:** Weighted proportions for all mental health, mental well-being, and school connectedness measures by academic year group.

	Year 8		Year 10		P-value
	%	95% CI	%	95% CI	
**Mental health and mental well-being**					
**Life satisfaction**					<0.001
*High*	21	18–23	12	10–14	
*Average*	44	42–46	43	40–46	
Low	35	32–38	45	41–49	
**Depression**					0.022
*No depression*	53	49–57	48	43–53	
*Probable depression*	16	14–17	16	15–18	
*Severe depression*	31	29–34	36	32–40	
**Anxiety**					0.014
*None to low*	42	38–47	39	35–43	
*Mild*	25	23–27	24	22–26	
*Moderate*	18	16–20	17	15–19	
*Severe*	15	14–17	20	16–23	
**Mental well-being**					<0.001
*High*	18	16–21	14	12–16	
*Average*	45	44–47	46	43–49	
*Low*	17	16–18	19	17–20	
*Poor*	19	17–21	22	19–25	
**Loneliness (No one to talk to)**					0.075
*Hardly*	46	44–49	43	40–46	
*Some*	35	32–37	36	34–37	
*Often*	19	17–21	21	19–24	
**Loneliness (Feeling left out)**					0.492
*Hardly*	42	39–44	40	37–43	
*Some*	38	36–40	40	38–42	
*Often*	20	18–22	20	18–23	
**Loneliness (Feeling alone)**					<0.001
*Hardly*	51	48–53	45	41–48	
*Some*	32	30–34	32	30–34	
*Often*	17	16–19	23	20–26	
**Loneliness (Scale of above three)**					
*Mean (SD)*	5.17	(0.06)	5.34	(0.08)	
**Loneliness (Direct question)**					<0.001
*Never*	19	17–21	16	14–17	
*Hardly ever*	29	27–31	26	23–28	
*Occasionally*	22	20–23	23	22–25	
*Sometimes*	20	18–22	21	20–23	
*Often*	11	9–12	14	12–17	
**School connectedness**					
**Feelings about school**					<0.001
*Like it a lot*	13	9–17	9	5–13	
*Like it a bit*	44	41–46	41	38–44	
*Don’t like it very much*	26	23–29	29	26–32	
*Don’t like it at all*	17	15–20	22	18–25	
**Schoolwork pressure**					<0.001
*Not at all*	14	11–17	8	7–10	
*A little*	34	31–37	28	26–30	
*Some*	25	23–27	26	24–28	
*A lot*	26	23–29	38	33–43	
**I feel like I belong in this school**					<0.001
*Agree*	52	46–57	43	37–49	
*Disagree*	48	43–54	57	51–63	
**I feel safe in this school**					0.002
*Agree*	55	50–61	48	40–57	
*Disagree*	45	39–50	52	43–60	
**Classmate support**					<0.001
*Very high*	18	13–22	10	7–14	
*High*	21	19–23	20	17–24	
*Low*	31	28–33	30	26–33	
*Very low*	31	26–36	40	34–45	
**Teacher support**					<0.001
*Very high*	24	20–27	16	13–19	
*High*	26	24–28	25	23–27	
*Low*	20	19–22	23	21–25	
*Very low*	30	26–33	36	32–39	
**Peer support**					0.007
*High*	47	43–51	45	41–49	
*Moderate*	24	22–27	29	27–31	
*Low*	29	26–32	26	22–29	

For the school connectedness measures, the largest difference was found in reports of schoolwork pressure; with 26% of Year 8 respondents reporting a lot of schoolwork pressure compared to 38% of Year 10 respondents. However, there were also large differences found for belonging in school and classmate support. Year 8 respondents were more likely to report a sense of belonging in school (52%), compared to Year 10 respondents (43%). Year 8 respondents were also more likely to report very high levels of classmate support (18%), compared to Year 10 respondents (10%).

### Mental health, mental well-being, and school connectedness by socioeconomic status

Table 5 shows the weighted mental health, mental well-being, and school connectedness measures by socioeconomic status. A significant difference for life satisfaction, symptoms of depression, anxiety, loneliness (having no one to talk to), and loneliness (direct question), by socioeconomic status, was found. The biggest differences were found for life satisfaction with 45% of the most deprived reporting low life satisfaction compared to 36% of the least deprived. Further, of the most deprived, 38% reported symptoms of severe depression compared to 30% of the least deprived.

**Table 5:** Weighted proportions for all mental health, mental well-being, and school connectedness measures by socioeconomic status.

	1 – Least		2		3		4		5 – Most		P-value
	%	95% CI	%	95% CI	%	95% CI	%	95% CI	%	95% CI	
**Mental health and mental well-being**											
**Life satisfaction**											0.022
*High*	17	15–19	18	16–20	15	12–18	15	12–19	17	13–21	
*Average*	47	43–50	42	39–46	45	41–49	41	37–44	39	35–42	
Low	36	33–40	40	36–43	41	35–46	44	40–48	45	40–49	
**Depression**											0.023
*No depression*	54	50–58	51	47–55	50	46–55	45	40–49	48	43–54	
*Probable depression*	16	14–18	16	14–18	17	13–20	17	14–20	14	12–17	
*Severe depression*	30	27–33	33	29–37	33	31–35	38	34–42	37	32–43	
**Anxiety**											0.040
*None to low*	44	41–48	40	36–44	40	35–45	37	32–42	39	32–47	
*Mild*	24	22–26	25	23–27	26	23–29	27	22–31	21	17–25	
*Moderate*	17	15–18	18	15–21	17	14–20	16	13–18	20	17–23	
*Severe*	15	13–17	18	15–21	17	14–20	21	17–25	20	16–23	
**Mental well-being**											0.049
*High*	17	14–21	17	14–20	16	12–19	13	10–16	17	13–21	
*Average*	47	44–50	47	45–50	44	40–47	44	41–48	41	34–47	
*Low*	17	16–19	16	15–18	19	16–22	19	16–22	19	16–23	
*Poor*	19	16–21	20	17–23	24	18–26	24	20–27	23	19–27	
**Loneliness (No one to talk to)**											<0.001
*Hardly*	49	47–51	45	42–48	45	41–49	38	34–42	41	37–45	
*Some*	33	32–35	33	30–36	35	33–37	43	41–46	37	34–39	
*Often*	18	16–19	22	20–25	20	16–23	19	15–23	22	19–26	
**Loneliness (Feeling left out)**											0.078
*Hardly*	42	39–46	40	38–42	43	39–47	36	31–41	39	34–45	
*Some*	40	37–43	39	37–41	36	34–39	41	38–44	39	34–43	
*Often*	18	16–20	21	17–24	21	18–24	23	17–28	22	19–25	
**Loneliness (Feeling alone)**											0.089
*Hardly*	50	47–52	48	44–52	47	43–52	43	37–48	48	43–53	
*Some*	32	30–34	32	30–33	31	28–33	36	32–39	30	25–35	
*Often*	18	16–20	20	17–23	22	19–25	22	18–26	22	19–25	
**Loneliness (Scale of above three)**											
*Mean (SD)*	5.11	(0.06)	5.29	(0.08)	5.26	(0.10)	5.47	(0.13)	5.37	(0.10)	
**Loneliness (Direct question)**											0.007
*Never*	16	15–17	17	15–19	19	17–22	16	13–19	19	15–24	
*Hardly ever*	30	28–32	27	24–29	26	23–28	26	23–29	25	22–28	
*Occasionally*	24	22–26	23	21–25	20	18–22	21	18–24	20	16–24	
*Sometimes*	19	16–21	21	19–23	22	19–24	23	19–28	21	19–23	
*Often*	11	9–13	12	9–15	13	11–16	14	11–16	15	12–18	
**School connectedness**											
**Feelings about school**											<0.001
*Like it a lot*	13	8–19	11	6–15	9	5–13	8	5–11	10	8–12	
*Like it a bit*	45	41–49	41	38–44	42	38–45	43	40–47	36	34–39	
*Don’t like it very much*	25	21–29	28	26–31	29	25–33	28	24–31	31	29–34	
*Don’t like it at all*	17	14–21	19	16–23	21	17–24	21	18–24	22	20–25	
**Schoolwork pressure**											0.067
*Not at all*	11	8–13	11	9–14	11	9–14	10	8–13	16	12–21	
*A little*	31	28–34	31	27–35	33	30–36	30	27–33	29	24–33	
*Some*	25	23–27	25	23–27	27	23–30	28	26–31	23	19–26	
*A lot*	33	29–37	33	28–37	29	25–33	31	28–35	32	29–36	
**I feel like I belong in this school**											<0.001
*Agree*	54	46–61	48	42–53	44	38–50	44	40–49	38	31–44	
*Disagree*	46	39–54	52	47–58	56	50–62	56	51–60	62	56–69	
**I feel safe in this school**											0.039
*Agree*	57	48–67	52	45–59	50	44–57	47	41–54	45	38–53	
*Disagree*	43	33–52	48	41–55	50	44–56	53	46–59	55	47–62	
**Classmate support**											0.265
*Very high*	14	9–19	14	11–17	15	10–20	14	11–18	14	9–18	
*High*	23	20–26	22	20–25	18	15–20	17	13–21	20	16–23	
*Low*	30	27–34	31	28–34	29	27–32	31	27–35	29	24–33	
*Very low*	33	26–40	33	28–39	38	34–42	37	32–43	38	31–45	
**Teacher support**											0.59
*Very high*	20	16–24	19	17–22	19	15–22	21	18–25	22	19–25	
*High*	26	24–28	27	24–30	25	23–28	25	22–28	25	20–29	
*Low*	22	20–24	20	18–23	21	19–23	24	21–26	23	20–26	
*Very low*	32	27–37	34	30–37	35	30–40	30	26–34	31	26–36	
**Peer support**											0.009
*High*	50	47–53	45	41–49	44	39–48	44	39–50	43	37–48	
*Moderate*	27	24–30	26	24–28	26	23–30	30	24–35	25	21–29	
*Low*	23	20–26	29	25–33	30	28–32	26	20–32	32	27–37	

Four of the seven school connectedness measures differed according to socioeconomic status. These measures were feelings about school, belonging in school, feeling safe in school, and peer support. Belonging in school had the largest variation by socioeconomic status where only 38% of the most deprived respondents reported a sense of belonging in school compared to 54% of the least deprived respondents. Deprivation was also associated with reported feelings of safety in school, with 45% of respondents from the most deprived group reporting feeling safe compared to 57% from the least deprived group. There were no large differences in reported levels of teacher support by socioeconomic status, but interestingly, slightly higher proportions of respondents from the most deprived groups felt supported by their teachers 22% versus 20% from the least deprived group.

### Regression analyses

Table 6 shows the regression outputs (Adjusted Odds Ratios (AOR), 95% CIs, associated p-values) for dependent and independent variables included in the final models. Forest plots of these regression analyses are available in S4 Fig. Gender, feeling a lot of schoolwork pressure, feeling safe in school, and classmate support were the four predictors that were highly predictive or protective of all the outcomes.

**Table 6:** Multivariate regression results for mental health and mental well-being.

	Life satisfaction	Depression	Anxiety	Mental well-being	Loneliness (No one to talk to)	Loneliness (Feeling left out)	Loneliness (Feeling alone)	Loneliness (Direct question)
	AOR 95% CI	AOR 95% CI	AOR 95% CI	AOR 95% CI	AOR 95% CI	AOR 95% CI	AOR 95% CI	AOR 95% CI
**Complete cases**	**3989**	**4000**	**4005**	**4014**	**4002**	**4009**	**4001**	
**Gender** **(Reference “*Boys*”)**	1.65 1.42-1.92 ***	3.27 2.84-3.76 ***	3.07 2.62-3.61 ***	2.03 1.74-2.36 ***	1.94 1.67-2.24 ***	1.90 1.67-2.15 ***	1.95 1.71-2.23 ***	2.13 1.84-2.46 ***
**Academic year group** **(Reference “*Year 8*”)**	1.37 1.18-1.59 ***	1.03 0.89-1.18	0.89 0.76-1.05	1.05 0.90-1.23	0.94 0.82-1.09	0.86 0.76-0.98 *	1.09 0.96-1.24	1.08 0.93-1.24
**Socioeconomic status** **(Continuous)**	0.98 0.95-1.01	0.97 0.95-1.00 *	0.97 0.94 −1.00 *	0.98 0.95-1.00	0.96 0.93-0.98 **	0.99 0.97-1.02	0.99 0.97-1.02	−1.01 0.98-1.03
**Feelings about school (Reference “*Like it a lot*”)**								
*Like it a bit*	1.34 0.98-1.85	1.30 0.99-1.73	1.40 1.00–2.04	1.60 1.15-2.28 **	–	–	–	–
*Don’t like it very much*	2.50 1.79-3.52 ***	1.89 1.40-2.56 ***	1.64 1.15-2.38 **	2.69 1.90-3.89 ***	–	–	–	–
*Don’t like it at all*	2.72 1.89-3.94 ***	2.30 1.66-3.22 ***	2.25 1.54-3.35 ***	4.06 2.78-6.02 ***	–	–	–	–
**Schoolwork pressure** **(Reference “*Not at all*”)**								
*A little*	1.15 0.86-1.54	1.48 1.12-1.98 **	1.42 1.00-2.04	1.13 0.83-1.54	1.58 1.24-2.04 ***	1.49 1.19-1.87 ***	1.46 1.14-1.88 **	1.55 1.21-2.00 ***
*Some*	1.46 1.09-1.97 *	2.33 1.76-3.12 ***	2.42 1.54-3.35 ***	1.70 1.26-2.33 ***	2.40 1.86-3.12 ***	2.30 1.82-2.91 ***	2.40 1.87-3.11 ***	2.72 2.11-3.52 ***
*A lot*	2.35 1.75–3.18 ***	4.53 3.40-6.08 ***	5.34 3.79-7.66 ***	2.77 2.04-3.79 ***	3.57 2.73-4.68 ***	3.06 (2.41–3.90) ***	3.91 3.03-5.08 ***	4.20 3.22-5.50 ***
**I feel like I belong in this school** **(Reference “*Agree*”)**	1.34 1.12-1.62 **	1.49 1.26-1.77 ***	1.47 1.21–1.79 ***	1.55 1.29-1.87 ***	1.50 1.26-1.81 ***	1.56 1.34-1.82 ***	1.68 1.43-1.96 ***	1.62 1.36-1.91 ***
**I feel safe in this school** **(Reference “*Agree*”)**	1.76 1.47-2.09 ***	2.03 1.72-2.39 ***	1.73 1.44–2.09 ***	1.37 1.15-1.64 ***	1.52 1.28-1.81 ***	1.50 1.30-1.75 ***	1.71 1.47-2.00 ***	1.55 1.31-1.83 ***
**Classmate support** **(Continuous)**	0.81 0.72–0.90 ***	0.73 0.66–0.81 ***	0.69 0.62–0.77 ***	0.81 0.72-0.91 ***	0.62 0.55-0.69 ***	0.63 0.58-0.69 ***	0.61 0.55-0.67 ***	0.60 0.54-0.67 ***
**Teacher support** **(Continuous)**	0.81 0.73–0.90 ***	0.88 0.80–0.98 *	–	0.85 0.77-0.95 **	0.80 0.72-0.88 ***	–	–	–
**Peer support** **(Continuous)**	0.91 0.88–0.95 ***	0.92 0.89 −0.96 ***	0.95 0.91–0.99 **	0.89 0.86-0.93 ***	0.93 0.90-0.97 ***	0.92 0.90-0.95 ***	0.91 0.88-0.94 ***	0.92 0.89-0.96 ***

*** < 0.001, ** < 0.01, * < 0.05.

Gender was an important predictor for all outcomes, with particularly stark differences in symptoms of depression (AOR 3.27 (CI: 2.84–3.76), Fig 2 in S4 Fig) and anxiety (AOR 3.07 (CI: 2.62–3.61), Fig 3, in S4 Fig), between girls and boys.

Academic year group was only an important predictor for life satisfaction (AOR 1.37 (CI: 1.18–1.59), Fig 1 in S4 Fig), with Year 10 students being more likely to report poor life satisfaction than Year 8 students. Academic year group was also somewhat important for loneliness (feeling left out); AOR 0.86 (CI: 0.76–0.98), with Year 8 respondents being more likely to report feeling left out (Fig 6 in S4 Fig).

Socioeconomic status was not highly correlated with any of the outcomes, however, higher socioeconomic status was a somewhat important protective factor in the loneliness (having no one to talk to) model (AOR 0.96 (CI: 0.93–0.98), Fig 5 in S4 Fig).

Not liking school very much or at all was a strong predictor for low life satisfaction, symptoms of depression, anxiety, and poor mental well-being (Figs 1-4 in S4 Fig), but was not included in any of the loneliness models. Not liking school at all was the strongest predictor for two of the eight outcomes; low life satisfaction (AOR 2.72 (CI: 1.89–3.94), Fig 1 in S4 Fig) and poor mental well-being (AOR 4.06 (CI: 2.78–6.02), Fig 4 in S4 Fig).

Feeling some or a lot of schoolwork pressure was an important predictor for all outcomes. Feeling a lot of schoolwork pressure was the strongest predictor for six of the eight models. These were symptoms of depression (AOR 4.53 (CI: 3.40–6.08), Fig 2 in S4 Fig), anxiety (AOR 5.34 (CI: 3.79–7.66), Fig 3 in S4 Fig), loneliness (no one to talk to) (AOR 3.57 (CI: 2.73–4.68), Fig 5 in S4 Fig), loneliness (feeling left out) (AOR 3.06 (CI: 2.41–3.90), Fig 6 in S4 Fig), loneliness (feeling alone) (AOR 3.91 (CI: 3.03–5.08), Fig 7 in S4 Fig), and loneliness (direct question) (AOR 4.20 (CI: 3.22–5.50), Fig 8 in S4 Fig). Feeling a lot of schoolwork pressure was consistently one of the most important predictors in all of the models.

Belonging in school, feeling safe in school, classmate support, and peer support were all important factors across the mental health and mental well-being outcomes. Classmate support was the key protective factor across all outcomes, most notably for the loneliness outcomes: loneliness (no one to talk to) (AOR 0.62 (CI: 0.55–0.69), Fig 5 in S4 Fig), loneliness (feeling left out) (AOR 0.63 (CI: 0.58–0.69), Fig 6 in S4 Fig), loneliness (feeling alone) (AOR 0.61 (CI:0.55–0.67), Fig 7 in S4 Fig), and loneliness (direct question) (AOR 0.60 (CI: 0.54–0.67), Fig 8 in S4 Fig).

While teacher support was not included in some of the models, it was an important protective factor for life satisfaction (AOR 0.81 (CI: 0.73–0.90), Fig 1 in S4 Fig) and loneliness (no one to talk to) (AOR 0.80 (CI: 0.72–0.88), Fig 5 in S4 Fig).

## Discussion

### Covid-19 pandemic context

All schools completed this survey during the 2019/2020 or 2020/2021 academic years, meaning they had all experienced Covid-19 pandemic-related disruption, including school closures, remote learning, phased reopening, student absences, and ongoing mitigation measures, in the previous or same academic year as these data were collected. These disruptions affected both the logistics of survey delivery (remote in two schools, classroom based in the remaining) and the experiences of the participating students. This may have contributed to the patterns of school connectedness observed and levels of loneliness found in our data. There is increasing evidence that pandemic related school closures increased loneliness and poor mental health in young people [61,62]. We also know from previous research that symptoms of anxiety decreased during the first pandemic lockdown and increased when students returned to school, this was more pronounced for students who had low levels of school connectedness pre-pandemic [62]. In our data, poor school connectedness was found across the sample, for instance just under half of respondents reported a sense of belonging in school, and just over half reported feeling safe in school. Inequalities were also found across the school connectedness measures, with large variations for belonging in school found for students from lower socioeconomic status groups.

### Mental health and mental well-being

The data reported here show that a sizeable minority of respondents in a sample of secondary schools in South West England reported poor mental health and mental well-being, with one third of respondents reporting severe symptoms of depression and one third reporting moderate and severe levels of anxiety. Further, there were notable inequalities in these outcomes by gender, academic year group, and socioeconomic status.

In England, in 2023, 32% of children aged 8–16 years had a probable or possible mental disorder and 39% of young people aged 17–19 years had a probable or possible mental disorder [5]. According to the European KIDSCREEN study (2008), the prevalence of poor mental health, among young people aged between eight and 18 years, was highest in the UK (borderline = 13%; abnormal = 10%) and Hungary (borderline = 11%; abnormal = 7%), with the lowest rates found in Germany (borderline = 7%; abnormal = 3%) and Switzerland (borderline = 6%; abnormal = 4%) [63].

Our data show stark differences in mental health and mental well-being outcomes between gender groups, with girls reporting poorer mental health and mental well-being than boys and nonbinary respondents also consistently reporting poorer outcomes than girls or boys. These differences in mental health and mental well-being outcomes between girls and boys are frequently found in the literature [13–17,64], and have been found cross-culturally and cross-nationally in a large analyses of mental health data from 73 different countries [12]. Although there is less evidence regarding non-cisgender adolescents, there is some evidence that there are notable mental health inequalities between them and cisgender adolescents [65,66]. Furthermore, our regression analysis demonstrated how girls were consistently more likely to report poor mental health and poor mental well-being outcomes, most notably for symptoms of depression and anxiety.

It is also well documented that students with a lower sense of connection to their school have poorer physical health, poorer mental health, and are more likely to take part in risk taking behaviour [67]. Additionally, there is good evidence that strong family relationships, peer support, and teacher support are protective of poor mental health [14,68]. Our data showed significant gender differences across all of the school connectedness measures, with girls reporting lower school connectedness than boys, and nonbinary respondents reporting lower school connectedness than girls and boys. School connectedness has been found to be a protective factor for mental health outcomes for both girls and boys, however, this relationship is more robust for girls than boys [29,66,69]. Our regression analysis revealed that not liking school at all, feeling a lot of schoolwork pressure, lacking a sense of belonging in school, and not feeling safe in school were all strong predictors for poor mental health and mental well-being. While classmate and peer support were important protective factors. Further, some studies have found girls to have higher levels of school connectedness than boys at younger ages but this declined over time [69,70].

The sizeable differences in mental health and school connectedness between nonbinary respondents and girls and boys highlights two vulnerable groups, but also provides an opportunity for directing school efforts to help and support these students. The school environment is associated with negative mental health outcomes for LGBTQ+ young people, often as a result of homophobic, biphobic, or transphobic bullying [71]. However, mental health outcomes for LGBTQ+ students can be improved with more supportive staff, support groups for LGBTQ+ students, inclusive syllabi and policies [71]. With regard to academic year group differences, our data showed for the majority of the mental health, mental well-being, and school connectedness outcomes, Year 10 respondents had poorer outcomes and felt less connected to their school than Year 8 respondents. However in the regression analyses, academic year group was only a significant predictor for poor life satisfaction. According to a large scale meta-analysis conducted in 2021 mental disorders in adulthood start early in the neurodevelopmental phases of life and peak by mid to late adolescence [72]. Additionally, according to a 2005 study [8], half of all mental health illnesses have manifested by the age of 14. Blakemore (2019), explains mental health illness is more likely to develop in adolescence rather than childhood as a result of neurodevelopmental processes which make the adolescent brain especially vulnerable to stressful or negative environmental events [64]. In addition, adolescence is marked by large increases in oestrogen in girls and testosterone in boys which is implicated in some of the observed mental health disparities found between girls and boys [64]. Thirdly, adolescents experience significant changes in their social environment, such as spending more time with friends and adjusting their self-perception to align with that of their peers [64]. Finally, Blakemore suggests adolescents are more inclined to be risk takers when with peers because of a heightened sensitivity to social exclusion [64]. Given these arguments, it is plausible that Year 8 respondents (aged 12–13 years), being in early adolescence, have likely developed fewer mental health challenges compared to Year 10 respondents. Further, Year 10 respondents are faced with increasing school and social pressure as they approach their GCSE exam year, possibly making them more susceptible to mental health difficulties [73]. Evidence also suggests that school connectedness is related to age, in that older students are less likely to feel connected to their school [29]. This likely explains the differences found in the prevalences of mental health and mental well-being and school connectedness by year group. This is also the probable reason for year group not being an important predictor in the regression analyses in the presence of school connectedness variables such as schoolwork pressure.

These differences highlight a need to develop and implement measures that provide additional mental health support for students as they progress through secondary school and that more effort is required to ensure students do not become alienated from school and are adequately supported to deal with mounting schoolwork pressure and for schools to consider how some of the pressure could be reduced.

There is good evidence to suggest that adolescents with lower socioeconomic status, lower perceived socioeconomic status, and lower parental education levels have more mental health problems compared to their peers with higher socioeconomic status indicators [20–23]. Similarly, our data revealed important differences between respondents living in the most and least deprived areas in terms of their mental health and mental well-being, most notably for life satisfaction and symptoms of depression. However, in the regression analysis, socioeconomic status was only somewhat associated with symptoms of depression, anxiety, and loneliness (having no one to talk to). Four (feelings about school, belonging in school, feeling safe in school, peer support) of the seven school connectedness measures differed across socioeconomic status categories while levels of perceived schoolwork pressure, classmate support, and teacher support were similar across respondents of different socioeconomic status.

Previous research has found that higher socioeconomic status was associated with higher levels of school connectedness [74]. This same study found that socioeconomic status was a moderator of the association between school connectedness and psychological distress [74].

Overall, our regression analyses found a strong relationship between school connectedness measures, such as feeling a lot of schoolwork pressure, and mental health and mental well-being outcomes.

### Policy implications

The large gender differences reported here suggests that schools need to recognise and consider that boys, girls, and nonbinary students likely experience and express school and mental health challenges differently. Secondly, given the importance of school connectedness in promoting good mental health, fostering school connectedness should be a central priority in all schools. According to the European KIDSCREEN study, poor social support was the strongest predictor of poor mental health, especially in the UK (OR= 6.05) compared to the overall odds for all included countries (OR= 3.85) [63]. It has been argued that the way schools affect student health is through their organisation, curriculum, and pedagogic practice which impacts students’ development [75]. The same paper argues that health promoting schools should focus on supporting students to achieve two key human capacities: practical reasoning (critical reflection) and affiliation (co-operation) with other people [75]. Policies and schools should prioritise whole school and health promoting approaches. Schools in England are subject to regular school inspections by the Office for Standards in Education, Children’s Services and Skills (Ofsted). Inspections cover areas such as quality of education, behaviour and attitudes, personal development of students and staff, and leadership and management. Inspections often influence the priorities of the schools, parents, and students [76] and provide a mechanism for encouraging schools to implement policies likely to foster greater connectedness and a sense of the school as a community.

### Future research

Given that these data were collected during and shortly after pandemic related disruptions, it would be important to observe current levels of school connectedness and mental health and well being in order to understand if the influence of the pandemic has settled or if the rates observed in our data remain. Future research should focus on how to address and improve school connectedness and school environments to improve mental health and mental well-being including potentially, focusing on addressing gender differences in levels of school connectedness. In addition, further details on gender identity could be explored. Future research could also explore mechanisms to reduce the gender gaps in mental health and mental well-being, especially in relation to nonbinary students. Future research could explore the mediating effect of bullying on levels of school connectedness, especially for different gender groups. The different facets of socioeconomic inequalities and how those impact different dimensions of mental health should be explored. Our findings revealed stark differences between socioeconomic status and school connectedness for certain measures, such as belonging in school, but not all measures, therefore the relationship and mechanisms behind these observations could be explored further as this is a potentially important avenue to addressing inequalities. SW-SHRN is designed as a systems intervention as it acknowledges the wider system in which schools operate. It aims to improve the health, well-being, and educational outcomes for young people by harnessing all the resources available within the system. This includes facilitating partnership working between schools, academic health researchers, and public health and/or education teams in local authorities to share knowledge and expertise.

### Strengths and limitations

This study benefits from a large sample of Year 8 and Year10 respondents in South West England, who are a good representative of the students from the 18 schools involved. Our findings are largely consistent with national and international literature, suggesting that the key associations and proposed points of intervention are relevant to other contexts with similar patterns—such as rising levels of poor adolescent mental health, gender and socioeconomic disparities, and low levels of school connectedness. Additionally, our paper benefits from using validated measures. However, all these measures are self-reported. This study adds to the growing body of literature around gender disparities in mental health outcomes and especially poor outcomes for gender minorities. The gender question included in our survey contained three categories, additional questions or an open-text field designed to capture more detail could shed more light on gender inequalities in mental health and mental well-being outcomes. This study also contributes to the growing body of evidence on the important role of schools, schoolwork pressure, social connection, on young people’s mental health and mental well-being, however, future work could further unpack the role that the school environment, including schoolwork pressure, plays and explore how to improve school connectedness especially for students most at risk of poor metal health and mental well-being.

The data used in this paper are cross-sectional, therefore we present correlations at a given point in time. These results cannot infer causality, which would be an important avenue to explore in future work. Additionally, slightly higher proportions of students from lower socioeconomic groups had missing data for some outcomes, which may introduce some bias in the effect estimates. Finally, these data only include students who were in school at the time, meaning students who were frequently absent or who were temporarily excluded may not be represented.

## Conclusion

This paper clearly demonstrates the high proportion of respondents in the early to mid teenage years who are experiencing poor mental health and poor mental well-being, such as low life satisfaction, symptoms of depression, anxiety, poor mental well-being, and loneliness. We have also shown the high proportion of respondents that are lacking a sense of connection with their schools. These findings are important as they highlight the large burden of mental health challenges faced by young people in South West England. These findings point to the urgent need for action to improve mental health in this age group, and particularly for those groups at higher risk of poor mental health and poor mental well-being, for their benefit and for the future health and economic security of the region. These groups include, girls, nonbinary students, students experiencing a lot of schoolwork pressure, and those feeling less connected to the schools., Researchers, policy makers, and schools can use this information to direct efforts at intervention and support.

## Supporting information

S1 Table Descriptive comparisons showing between school variation in school connectedness and mental health and well-being outcomes. (DOCX)

S2 Text Additional study method details. (DOCX)

S3 Table Frequencies and proportions across key measures for students with and without data on the student, teacher, and peer support measures. (DOCX)

S4 Fig Forest plots of regression analyses. (DOCX)
