# Supplementary material for: Prevalence of mental health, mental well-being, and school connectedness in UK secondary school students in 2021/2022: Results from the South West - School Health Research Network pilot study
Source: PLoS One. 2026 Aug 28;21(8):e0355874. doi: 10.1371/journal.pone.0355874 (PMC13524283; doi:10.1371/journal.pone.0355874)
Supplement: Supplementary file 1 [file pone.0355874.s001.docx]

# S1 Table

**Table 1. Descriptive comparisons showing between school variation in school connectedness and mental health and well-being outcomes**

|  | **Minimum** | **Maximum** | **Difference** |
| --- | --- | --- | --- |
| **Mental health** | | | |
| **Life satisfaction** |  |  |  |
| *High* | 9.8 | 24.5 | 14.7 |
| *Average* | 33.7 | 55.6 | 21.9 |
| Low | 20.1 | 50 | 29.9 |
| **Depression** |  |  |  |
| *No depression* | 31.9 | 68.6 | 36.7 |
| *Probable depression* | 9.6 | 21 | 11.4 |
| *Severe depression* | 21.8 | 54.2 | 32.4 |
| **Anxiety** |  |  |  |
| *None to low* | 28.8 | 61.2 | 32.4 |
| *Mild* | 16.6 | 33.1 | 16.5 |
| *Moderate* | 12.5 | 22.6 | 10.1 |
| *Severe* | 8.5 | 32.9 | 24.4 |
| **Mental well-being** |  |  |  |
| *High* | 8.8 | 33.1 | 24.3 |
| *Average* | 31.2 | 49.5 | 18.3 |
| *Low* | 12.3 | 29.2 | 16.9 |
| *Poor* | 7.3 | 37.6 | 30.3 |
| **Loneliness (No one to talk to)** |  |  |  |
| *Hardly* | 29.3 | 57.6 | 28.3 |
| *Some* | 28.6 | 41 | 12.4 |
| *Often* | 13.7 | 32 | 18.3 |
| **Loneliness (Feeling left out)** |  |  |  |
| *Hardly* | 21.3 | 57.1 | 35.8 |
| *Some* | 26.8 | 46.3 | 19.5 |
| *Often* | 12.6 | 36 | 23.4 |
| **Loneliness (Feeling alone)** |  |  |  |
| *Hardly* | 30.7 | 61.5 | 30.8 |
| *Some* | 25.6 | 40.4 | 14.8 |
| *Often* | 12.8 | 38.7 | 25.9 |
| **Loneliness (Direct question)** |  |  |  |
| *Never* | 9.6 | 24.1 | 14.5 |
| *Hardly ever* | 19.8 | 38.2 | 18.4 |
| *Occasionally* | 17.9 | 27.5 | 9.6 |
| *Sometimes* | 14.1 | 28.5 | 14.4 |
| *Often* | 6.4 | 24.7 | 18.3 |
| **School connectedness** | | | |
| **Feelings about school** |  |  |  |
| *Like it a lot* | 6.2 | 39.9 | 33.7 |
| *Like it a bit* | 28.6 | 55.7 | 27.1 |
| *Don’t like it very much* | 9.5 | 33.7 | 24.2 |
| *Don’t like it at all* | 3 | 33.8 | 30.8 |
| **Schoolwork pressure** |  |  |  |
| *Not at all* | 5.7 | 21.7 | 16 |
| *A little* | 19.4 | 43.5 | 24.1 |
| *Some* | 19.7 | 30.6 | 10.9 |
| *A lot* | 15.2 | 47.2 | 32 |
| **I feel like I belong in this school** |  |  |  |
| *Disagree* | 19.1 | 72.5 | 53.4 |
| **I feel safe in this school** |  |  |  |
| *Disagree* | 10.8 | 64.7 | 53.9 |
| **Classmate support** |  |  |  |
| *Very high* | 8.2 | 39.8 | 31.6 |
| *High* | 14.4 | 30.9 | 16.5 |
| *Low* | 21.6 | 35.8 | 14.2 |
| *Very low* | 7.7 | 46.2 | 38.5 |
| **Teacher support** |  |  |  |
| *Very high* | 11 | 35.6 | 24.6 |
| *High* | 21.4 | 34.3 | 12.9 |
| *Low* | 15.1 | 26.6 | 11.5 |
| *Very low* | 19 | 46.2 | 27.2 |
| **Peer support** |  |  |  |
| *High* | 35.5 | 63.8 | 28.3 |
| *Moderate* | 17.3 | 31.9 | 14.6 |
| *Low* | 17.6 | 38.1 | 20.5 |
