# Supplementary material for: Prevalence of mental health, mental well-being, and school connectedness in UK secondary school students in 2021/2022: Results from the South West - School Health Research Network pilot study
Source: PLoS One. 2026 Aug 28;21(8):e0355874. doi: 10.1371/journal.pone.0355874 (PMC13524283; doi:10.1371/journal.pone.0355874)
Supplement: Supplementary file 2 [file pone.0355874.s002.docx]

# S2 Text

# Measures

## Demographics

Gender response options included; girl, boy, and neither word describes me. To enhance readability the term nonbinary was used throughout the manuscript. The academic year group, date of birth, and residential postcode of each student was provided by the school. Date of birth was then used to calculate the students’ age at the time that they completed the survey.

The postcodes provided by the school were used to obtain the Index of Multiple Deprivation (IMD), which is a measure of relative deprivation across small areas [1]. IMD is a combined measure from seven facets such as income and health [1]. IMD was used as an indicator of socioeconomic status and was included as an ordinal variable in the prevalence component of this study and was included as a continuous variable in the regression analysis. The continuous variable ranges from 1 to 10 with 1 indicating the most deprived.

For ethnicity, students were asked how they would describe themselves and were provided with six options (White, Mixed/Multiple ethnic groups, Asian/Asian British, Black/African/Caribbean/Black British, Arab, Any other ethnic group, I do not want to answer). These categories are those recommended for England by the Office for National Statistics [2].

Self-reported disability was measured by asking the students if their day-to-day activities were limited because of a health problem or disability which had lasted or was expected to last for at least 12 months. If a student responded ‘yes’ they were further asked if this disability affected their attendance or participation at school.

Household structure was explored by asking students how many adults and siblings live in their household. For the number of adults, response options ranged from 1 to 5 or more, while for siblings, response options ranged from 0 to 5 or more.

## Mental health

Life satisfaction was measured using the validated Cantril Scale, which is also used in the HBSC questionnaire [3]. Responses range from 0 (worst possible life) to 10 (best possible life). This variable can be explored continuously or as a categorical variable with three possible levels; low (0-6), average (7-8), and high (9-10). Here we explored this variable as a categorical variable.

Depression was measured using the Short Mood and Feelings Questionnaire (SMFQ) [4], this 13 item questionnaire being derived from the longer 33 item version, Mood and Feelings Questionnaire (MFQ). Both are used as screening tools for the identification of depressive symptoms in children and adolescents. The SMFQ is a valid measure in both clinical and community settings [4, 5]. Students were asked how they have been feeling over the past two weeks, and they select on a scale of 0 = ‘Not true’, 1 = ‘Sometimes’ or 2 = ‘True’ for each statement. Scores range from 0 to 26, a score of 7 or less indicates no probable depression, while a score between 8 and 11 indicates probable depression, and a score of 12 or higher indicates more severe symptoms of depression.

Anxiety was measures using the Generalised Anxiety Disorder – 7 (GAD-7) scale [6], which consists of seven items that are answered on a scale of 0 to 3 (‘Not at all’, ‘Several days’, ‘More than half the days’, ‘Nearly every day’). This tool has been validated among adults and adolescents [7]. Scores can range from 0 to 21, a range from 0-4 indicates ‘None to low anxiety’, 5-9 ‘Mild’, 10-14 ‘Moderate’, and more than 15 ‘Severe’.

Mental well-being was measured using the 7-item Short Warwick-Edinburgh Mental Well-being Scale (SWEMWBS) which is a shortened version of the 14 item Warwick-Edinburgh Mental Well-being Scale (WEMWBS). SWEMWBS was shortened, due to redundancy and improved scaling, however the correlation between the short and the long scales is high [8]. Students respond to the seven statements on a five-point Likert scale (‘None of the time’, ‘Rarely’, ‘Some of the time’, ‘Often’, ‘All of the time’), which are scored from 1 to 5, respectively. Total scores range from seven to 35 and the summed scored is broken into 4 categories; Poor (<=17), Low (18- 20), Average (21- 27), High mental well-being (>=28).

To explore loneliness we used the measures, designed for children aged between 10 and 15 years, recommended by the Office for National Statistics [9]. They specifically recommend using four questions, three of which form part of a loneliness scale and one direct question. There are three response categories for the scale questions (‘Hardly ever or never’, ‘Some of the Time’, ‘Often’) and five for the direct (‘Often/always’, ‘Some of the time’, ‘Occasionally’, ‘Hardly ever’, ‘Never’). For the scale questions responses are assigned a score between one and three, respectively, resulting in a total score between three and nine. There are no categories derived from the total score, but it can be used to compare different groups or populations. All four questions can also be explored independently.

## Social and School connectedness

Feelings about school and schoolwork pressure were measured using two single measures taken from the HBSC questionnaire [10, 11]. Students were asked how they felt about school at present, with four responses ranging from ‘I like it a lot’ to ‘I do not like it at all’. Similarly, schoolwork pressure was measured by asking how pressured they felt by schoolwork. They were provided with four responses ranging from ‘Not at all’ to ‘A lot’.

Belonging and feeling safe in school were measured using single questions from the HBSC questionnaire. Students were provided with two statements ‘I feel like I belong in this school’ and ‘I feel safe in this school’ and were provided with a five-point Likert scale of options ranging from ‘Strongly agree’ to ‘Strongly disagree’. Responses were binarised into 'Agree' (including ‘Strongly agree’ and ‘Agree’) and 'Disagree' (including ‘Neither agree nor disagree’, ‘Disagree’, and ‘Strongly disagree’)

Classmate and teacher support were measured using three and four questions respectively, to form two composite scales. Students were provided with three statements for each scale, such as ‘The students in my class(es) enjoy being together’ or ‘I feel my teachers accept me as I am’. They then selected how much they agree with each on a five-point scale ranging from ‘Strongly agree’ to ‘Strongly disagree’. The scores for each scale were summed and divided by three, with final scores ranging between one and five where 5 indicated good support. Each set is then ranked in four quantiles [12]. The student support quantiles were; quantile 1 [1-3], quantile 2 [3.1-3.67], quantile 3 [3.67-4], quantile 4 [4.1-5]. While the resultant teacher support quantiles were; quantile 1 [1-3], quantile 2 [3.1-3.5], quantile 3 [3.5-4], quantile 4 [4.1-5]. Both were assigned the following labels;; quantile 1 [very low support], quantile 2 [low support], quantile 3 [high support], quantile 4 [very high support]. Both the classmate and teacher support scales were from the HBSC questionnaire.

Peer support was measured using the peer support subset of questions from the Multidimensional Scale of Perceived Social Support (MSPSS), the reliability and validity of this scale has been widely tested [13]. The peer support subscale includes four questions for which the students confirm their agreement or disagreement on a scale of one to seven. These are summed and divided by four, the total is then categorised into; ‘Low’ (<3), ‘Moderate’ (3 - <5), and ‘High’ (>= 5) [14].

# Regression analysis

All mental health and mental well-being outcomes were categorical, and were modelled using Ordinal Logistic Regression (OLR). Prior to running the OLR models all assumptions were checked. One of the assumptions checked was the proportional odds assumption, which means that the relationship is the same for all cut points of the ordinal outcome. This is tested by creating dummy variables of each pair of outcome categories, which are them then modelled with the predictors. Estimates from these models and from the complete ordinal variable model are compared. The assumption is violated if these estimates are not comparable. In these analyses the proportional odd assumption was violated, for life satisfaction, anxiety, mental well-being, loneliness- No one to talk to, and loneliness-direct. For these outcome variables the most balanced binary form of the variable was used (Table 1).

| **Variable** | **Categorical** | **Binary** |
| --- | --- | --- |
| **Life satisfaction** | 1. High 2. Average 3. Low | 1. Average + High 2. Low |
| **Anxiety** | 1. None to low 2. Mild 3. Moderate 4. Severe | 1. None to low + Mild 2. Moderate + Severe |
| **Mental well-being** | 1. Poor 2. Low 3. Average 4. High | 1. Poor + Low 2. Average + High |
| **Loneliness - No one to talk to** | 1. Hardly ever 2. Some of the time 3. Often | 1. Hardly ever 2. Some of the time + Often |
| **Loneliness - Direct** | 1. Never 2. Hardly ever 3. Occasionally 4. Sometime of the time 5. Often | 1. Never + Hardly ever 2. Occasionally + Sometime of the time + Often |

## Backward elimination with model comparison

Backwards elimination was used to remove non-significant (p-value >= 0.05) school connectedness variables from each model. Gender, academic year group, socioeconomic status were not removed regardless of their significance, allowing them to be controlled for in the final models. Each resultant model with an excluded variable was compared to the previous model using ANOVA (analysis of deviance), a p-value of < 0.05 indicated that a variable had significant contribution to the overall model (despite its correlation with the outcome) and therefore was not excluded from the final model.

# References

1.	Ministry of Housing CLG. English indices of deprivation 2019 2019 [Available from: <https://www.gov.uk/government/statistics/english-indices-of-deprivation-2019>.

2.	Office for National Statistics. Ethnic group, national identity and religion. Measuring equality: A guide for the collection and classification of ethnic group, national identity and religion data in the UK.: Open Government Licence v3.0; [Available from: <https://www.ons.gov.uk/methodology/classificationsandstandards/measuringequality/ethnicgroupnationalidentityandreligion>.

3.	Levin KA, Currie C. Reliability and Validity of an Adapted Version of the Cantril Ladder for Use with Adolescent Samples. Social Indicators Research. 2014;119(2):1047-63.

4.	Angold A, Costello EJ, Messer SC, Pickles A. Development of a short questionnaire for use in epidemiological studies of depression in children and adolescents. Int J Methods Psychiatr Res. 1995.

5.	Sharp C, Goodyer IM, Croudace TJ. The Short Mood and Feelings Questionnaire (SMFQ): A Unidimensional Item Response Theory and Categorical Data Factor Analysis of Self-Report Ratings from a Community Sample of 7-through 11-Year-Old Children. J Abnorm Child Psychol. 2006;34(3):365-77.

6.	Spitzer RL, Kroenke K, Williams JBW, Löwe B. A Brief Measure for Assessing Generalized Anxiety Disorder: The GAD-7. Arch Intern Med. 2006;166(10):1092-7.

7.	Romano I, Ferro MA, Patte KA, Leatherdale ST. Measurement Invariance of the GAD-7 and CESD-R-10 Among Adolescents in Canada. J Pediatr Psychol. 2021;47(5):585-94.

8.	Stewart-Brown S, Tennant A, Tennant R, Platt S, Parkinson J, Weich S. Internal construct validity of the Warwick-Edinburgh Mental Well-being Scale (WEMWBS): a Rasch analysis using data from the Scottish Health Education Population Survey. Health and Quality of Life Outcomes. 2009;7(1):15.

9.	Office for Nation Statistics. Measuring loneliness: guidance for use of the national indicators on surveys [updated 2018. Available from: <https://www.ons.gov.uk/peoplepopulationandcommunity/wellbeing/methodologies/measuringlonelinessguidanceforuseofthenationalindicatorsonsurveys>.

10.	Currie C, Nic Gabhainn S, Godeau E, Committee IHNC. The Health Behaviour in School-aged Children: WHO Collaborative Cross-National (HBSC) study: origins, concept, history and development 1982–2008. International journal of public health. 2009;54(Suppl 2):131-9.

11.	Currie C, Zanotti C, Morgan A, Currie D, de Looze M, et al. Social determinants of health and well-being among young people: Health Behaviour in School-aged Children (‎HBSC)‎ study: international report from the 2009/2010 survey. World Health Organization. Regional Office for Europe.; 2012 2012.

12.	García-Moya I, Brooks FM, Spencer NH. School-level factors associated with teacher connectedness: a multilevel analysis of the structural and relational school determinants of young people’s health. Journal of Public Health. 2017;40(2):366-74.

13.	Dahlem NW, Zimet GD, Walker RR. The multidimensional scale of perceived social support: a confirmation study. J Clin Psychol. 1991;47(6):756-61.

14.	Zimet G. Multidimensional Scale of Perceived Social Support (MSPSS) - Scale Items and Scoring Information 2016 [Available from: <https://www.researchgate.net/publication/311534896_Multidimensional_Scale_of_Perceived_Social_Support_MSPSS_-_Scale_Items_and_Scoring_Information>.
