# Supplementary material for: Prevalence of mental health, mental well-being, and school connectedness in UK secondary school students in 2021/2022: Results from the South West - School Health Research Network pilot study
Source: PLoS One. 2026 Aug 28;21(8):e0355874. doi: 10.1371/journal.pone.0355874 (PMC13524283; doi:10.1371/journal.pone.0355874)
Supplement: Supplementary file 3 [file pone.0355874.s003.docx]

# S3 Tables

**Table 1. Frequencies and proportions across key measures for students with and without data on the student support measure**

|  | **Not missing**  **n (%)** | **Missing**  **n (%)** | **P-value** |
| --- | --- | --- | --- |
| **Total** | **4642** | **551** |  |
| **Academic year group:** |  |  |  |
| *Year 10* | 2234 (48.2) | 211 (38.3) | <0.001 |
| **Gender** |  |  | 0.257 |
| *Boys* | 2343 (51.2) | 282 (53.3) |  |
| *Girls* | 2096 (45.8) | 226 (42.7) |  |
| *Nonbinary* | 139 ( 3.0) | 21 ( 4.0) |  |
| **Socioeconomic status** |  |  | <0.001 |
| *1 (Least deprived)* | 1453 (31.6) | 140 (25.6) |  |
| *2* | 1242 (27.0) | 152 (27.8) |  |
| *3* | 883 (19.2) | 76 (13.9) |  |
| *4* | 545 (11.8) | 87 (15.9) |  |
| *5 (Most deprived)* | 482 (10.5) | 91 (16.7) |  |
| **Life satisfaction** |  |  | <0.001 |
| *High* | 767 (16.8) | 79 (16.1) |  |
| *Average* | 2017 (44.2) | 175 (35.6) |  |
| *Low* | 1781 (39.0) | 237 (48.3) |  |
| **Depression** |  |  | <0.001 |
| *No depression* | 2368 (51.7) | 186 (40.2) |  |
| *Probable depression* | 723 (15.8) | 87 (18.8) |  |
| *Severe depression* | 1485 (32.5) | 190 (41.0) |  |
| **Anxiety** |  |  | 0.514 |
| *None to low* | 1896 (41.3) | 168 (37.8) |  |
| *Mild* | 1115 (24.3) | 113 (25.5) |  |
| *Moderate* | 783 (17.1) | 84 (18.9) |  |
| *Severe* | 792 (17.3) | 79 (17.8) |  |
| **Mental well-being** |  |  | 0.001 |
| *High* | 785 (17.1) | 47 (10.4) |  |
| *Average* | 2075 (45.2) | 207 (45.8) |  |
| *Low* | 812 (17.7) | 82 (18.1) |  |
| *Poor* | 923 (20.1) | 116 (25.7) |  |
| **Loneliness (No one to talk to)** |  |  | 0.245 |
| *Hardly* | 2077 (45.3) | 180 (41.1) |  |
| *Some* | 1603 (34.9) | 164 (37.4) |  |
| *Often* | 909 (19.8) | 94 (21.5) |  |
| **Loneliness (Feeling left out)** |  |  | 0.138 |
| *Hardly* | 1898 (41.3) | 165 (37.2) |  |
| *Some* | 1785 (38.8) | 176 (39.6) |  |
| *Often* | 914 (19.9) | 103 (23.2) |  |
| **Loneliness (Feeling alone)** |  |  | 0.019 |
| *Hardly* | 2219 (48.4) | 186 (42.4) |  |
| *Some* | 1437 (31.3) | 165 (37.6) |  |
| *Often* | 930 (20.3) | 88 (20.0) |  |
| **Loneliness (Direct question)** |  |  | 0.003 |
| *Never* | 784 (17.1) | 94 (20.8) |  |
| *Hardly ever* | 1281 (27.9) | 95 (21.0) |  |
| *Occasionally* | 1036 (22.6) | 91 (20.1) |  |
| *Sometimes* | 915 (19.9) | 110 (24.3) |  |
| *Often* | 573 (12.5) | 63 (13.9) |  |

**Table 2. Frequencies and proportions across key measures for students with and without data on the teacher support measure**

| **Teacher support** | **Not missing**  **n (%)** | **Missing**  **n (%)** | **P-value** |
| --- | --- | --- | --- |
| **Total** | **4618** | **575** |  |
| **Academic year group:** |  |  |  |
| *Year 10* | 2228 (48.3) | 217 (37.7) | <0.001 |
| **Gender** |  |  |  |
| *Boys* | 2347 (51.5) | 278 (50.3) |  |
| *Girls* | 2070 (45.5) | 252 (45.6) |  |
| *Nonbinary* | 137 ( 3.0) | 23 ( 4.2) |  |
| **Socioeconomic status** |  |  | <0.001 |
| *1 (Least deprived)* | 1443 (31.5) | 150 (26.3) |  |
| *2* | 1239 (27.1) | 155 (27.1) |  |
| *3* | 870 (19.0) | 89 (15.6) |  |
| *4* | 547 (11.9) | 85 (14.9) |  |
| *5 (Most deprived)* | 481 (10.5) | 92 (16.1) |  |
| **Life satisfaction** |  |  | 0.001 |
| *High* | 759 (16.7) | 87 (17.0) |  |
| *Average* | 2007 (44.2) | 185 (36.1) |  |
| *Low* | 1777 (39.1) | 241 (47.0) |  |
| **Depression** |  |  | <0.001 |
| *No depression* | 2348 (51.6) | 206 (42.4) |  |
| *Probable depression* | 724 (15.9) | 86 (17.7) |  |
| *Severe depression* | 1481 (32.5) | 194 (39.9) |  |
| **Anxiety** |  |  | 0.863 |
| *None to low* | 1876 (41.2) | 188 (39.6) |  |
| *Mild* | 1105 (24.3) | 123 (25.9) |  |
| *Moderate* | 785 (17.2) | 82 (17.3) |  |
| *Severe* | 789 (17.3) | 82 (17.3) |  |
| **Mental well-being** |  |  | 0.021 |
| *High* | 776 (17.0) | 56 (11.9) |  |
| *Average* | 2065 (45.1) | 217 (46.2) |  |
| *Low* | 810 (17.7) | 84 (17.9) |  |
| *Poor* | 926 (20.2) | 113 (24.0) |  |
| **Loneliness (No one to talk to)** |  |  | 0.372 |
| *Hardly* | 2047 (44.9) | 210 (45.1) |  |
| *Some* | 1614 (35.4) | 153 (32.8) |  |
| *Often* | 900 (19.7) | 103 (22.1) |  |
| **Loneliness (Feeling left out)** |  |  | 0.520 |
| *Hardly* | 1878 (41.1) | 185 (39.4) |  |
| *Some* | 1780 (38.9) | 181 (38.5) |  |
| *Often* | 913 (20.0) | 104 (22.1) |  |
| **Loneliness (Feeling alone)** |  |  | 0.516 |
| *Hardly* | 2194 (48.1) | 211 (45.4) |  |
| *Some* | 1445 (31.7) | 157 (33.8) |  |
| *Often* | 921 (20.2) | 97 (20.9) |  |
| **Loneliness (Direct question)** |  |  | 0.032 |
| *Never* | 777 (17.0) | 101 (21.3) |  |
| *Hardly ever* | 1265 (27.7) | 111 (23.4) |  |
| *Occasionally* | 1034 (22.6) | 93 (19.6) |  |
| *Sometimes* | 919 (20.1) | 106 (22.3) |  |
| *Often* | 572 (12.5) | 64 (13.5) |  |

**Table 3. Frequencies and proportions across key measures for students with and without data on the peer support measure**

| **Peer support** | **Not missing**  **n (%)** | **Missing**  **n (%)** | **P-value** |
| --- | --- | --- | --- |
| **Total** | **4494** | **699** |  |
| **Academic year group:** |  |  | <0.001 |
| *Year 10* | 2168 (48.3) | 277 (39.6) |  |
| **Gender** |  |  | 0.284 |
| *Boys* | 2259 (51.0) | 366 (53.7) |  |
| *Girls* | 2031 (45.9) | 291 (42.7) |  |
| *Nonbinary* | 136 ( 3.1) | 24 ( 3.5) |  |
| **Socioeconomic status** |  |  | <0.001 |
| *1 (Least deprived)* | 1399 (31.4) | 194 (28.1) |  |
| *2* | 1218 (27.3) | 176 (25.5) |  |
| *3* | 859 (19.3) | 100 (14.5) |  |
| *4* | 524 (11.7) | 108 (15.6) |  |
| *5 (Most deprived)* | 460 (10.3) | 113 (16.4) |  |
| **Life satisfaction** |  |  | 0.004 |
| *High* | 735 (16.6) | 111 (17.5) |  |
| *Average* | 1955 (44.2) | 237 (37.4) |  |
| *Low* | 1732 (39.2) | 286 (45.1) |  |
| **Depression** |  |  | 0.039 |
| *No depression* | 2273 (51.3) | 281 (46.1) |  |
| *Probable depression* | 697 (15.7) | 113 (18.5) |  |
| *Severe depression* | 1459 (32.9) | 216 (35.4) |  |
| **Anxiety** |  |  | 0.347 |
| *None to low* | 1821 (41.0) | 243 (41.0) |  |
| *Mild* | 1074 (24.2) | 154 (26.0) |  |
| *Moderate* | 760 (17.1) | 107 (18.1) |  |
| *Severe* | 783 (17.6) | 88 (14.9) |  |
| **Mental well-being** |  |  | 0.013 |
| *High* | 757 (17.0) | 75 (12.5) |  |
| *Average* | 2002 (45.0) | 280 (46.5) |  |
| *Low* | 791 (17.8) | 103 (17.1) |  |
| *Poor* | 895 (20.1) | 144 (23.9) |  |
| **Loneliness (No one to talk to)** |  |  | 0.385 |
| *Hardly* | 1982 (44.7) | 275 (46.7) |  |
| *Some* | 1575 (35.5) | 192 (32.6) |  |
| *Often* | 881 (19.9) | 122 (20.7) |  |
| **Loneliness (Feeling left out)** |  |  | 0.289 |
| *Hardly* | 1805 (40.6) | 258 (43.7) |  |
| *Some* | 1747 (39.2) | 214 (36.3) |  |
| *Often* | 899 (20.2) | 118 (20.0) |  |
| **Loneliness (Feeling alone)** |  |  | 0.659 |
| *Hardly* | 2125 (47.9) | 280 (47.7) |  |
| *Some* | 1407 (31.7) | 195 (33.2) |  |
| *Often* | 906 (20.4) | 112 (19.1) |  |
| **Loneliness (Direct question)** |  |  | 0.200 |
| *Never* | 754 (17.0) | 124 (20.7) |  |
| *Hardly ever* | 1213 (27.3) | 163 (27.3) |  |
| *Occasionally* | 1006 (22.6) | 121 (20.2) |  |
| *Sometimes* | 909 (20.5) | 116 (19.4) |  |
| *Often* | 562 (12.6) | 74 (12.4) |  |
