# Supplementary material for: Prevalence of mental health, mental well-being, and school connectedness in UK secondary school students in 2021/2022: Results from the South West - School Health Research Network pilot study
Source: PLoS One. 2026 Aug 28;21(8):e0355874. doi: 10.1371/journal.pone.0355874 (PMC13524283; doi:10.1371/journal.pone.0355874)
Supplement: Supplementary file 4 [file pone.0355874.s004.docx]

# S4. Figures

Below are the forest plots depicting variables associated with increased risk of mental health and mental well-being outcomes. The forest plots show the adjusted odds ratios and corresponding 95% confidence intervals (CIs) for the included variables from the multivariate regression models, controlling for gender, academic year group, and socioeconomic status. Variables with CIs that cross the zero line are not statistically significant. P-values are indicated as follows: *** <0.001 , ** <0.01 , * <0.05. Point estimates that fall below 1 are depicted in red, while those above 1 are depicted in blue.

Fig 1. Forest plot depicting multivariate regression model for life satisfaction.

Fig 2. Forest plot depicting multivariate regression model for depression.

Fig 3. Forest plot depicting multivariate regression model for anxiety.

Fig 4. Forest plot depicting multivariate regression model for mental well-being.

Fig 5. Forest plot depicting multivariate regression model for loneliness (no one to talk to).

Fig 6. Forest plot depicting multivariate regression model for loneliness (feeling left out).

Fig 7. Forest plot depicting multivariate regression model for loneliness (feeling alone).

Fig 8. Forest plot depicting multivariate regression model for loneliness (direct).
