# Supplementary material for: Prevalence of mental health, mental well-being, and school connectedness in UK secondary school students in 2021/2022: Results from the South West - School Health Research Network pilot study
Source: PLoS One. 2026 Aug 28;21(8):e0355874. doi: 10.1371/journal.pone.0355874 (PMC13524283; doi:10.1371/journal.pone.0355874)
Supplement: Supplementary file 5 [file pone.0355874.s005.docx]

**Reviewer Comments**

Manuscript Title: Prevalence of mental health, mental well-being, and school connectedness in UK secondary school students: results from the South West - School Health Research Network pilot study.

Manuscript Number: PONE-D-25-55322

**Overall Comments:** The manuscript is well-written and logically organized. However, addressing the following minor points will strengthen the clarity and rigor of the work.

**Specific Comments:**

Abstract

Please include key findings from the regression analysis, providing precise estimates (e.g., AOR with CI) alongside the existing descriptive statistics.

Methods

Data Collection Rationale: Provide a brief justification (2–3 lines) for the use of self-reported data.

Data Quality & Non-Response: Detail how data quality was maintained, specifically addressing non-response rates and the characteristics of those who did not participate.

Participant Understanding: Explain how you ensured respondents understood the questions in the self-administered format. Was there a mechanism for participants to contact the research team for clarification or further queries?

Variables: Clearly define the primary explanatory variables and the covariates included in the regression models.

Discussion:

Limitations: Expand on the study's limitations, particularly those inherent to cross-sectional designs, and discuss how these could be addressed or mitigated in future research.

Policy Implications: Introduce a dedicated subsection discussing the policy implications derived directly from your findings.
