# Supplementary material for: Prevalence of mental health, mental well-being, and school connectedness in UK secondary school students in 2021/2022: Results from the South West - School Health Research Network pilot study
Source: PLoS One. 2026 Aug 28;21(8):e0355874. doi: 10.1371/journal.pone.0355874 (PMC13524283; doi:10.1371/journal.pone.0355874)
Supplement: Supplementary file 6 [file pone.0355874.s006.docx]

**Response to reviewers**

***Note from the Authors of this paper:***

*General feedback and suggestions or comments from reviewers are detailed below. Under each comment or suggestion we provide a detailed response, this is indented and italicised. The page and line numbers correspond to the document with tracked changes.*

*We would like to the take the opportunity to thank the reviewers for their time and their valuable feedback which has certainly strengthened the overall paper.*

**Reviewer #1:**

Thank you for giving me the opportunity to review your work.

School connectedness and belonging are important to examine across all schools.

This reviewer gives you much support on the current work and to continue this work going forward.

See below for suggestions/comments.

1. Abstract- line 47- correct deprive to deprived

*The abstract has been updated to reflect the additional analyses undertaken.*

2. On line 86 you use a JOSH reference from 2004. Why did you choose this for your definition of school connectedness? Is there something addressing this more recent?

*We have amended our definition, drawing on works from 2020 and 2022. The core concept of this definition has not been changed and therefore does not impacted any further interpretation within the paper. This change reflects that the conceptualisation of school connectedness has not shifted much over the last 2 decades. The updated definition now reads:*

*Pg 5, Ln 113: “School connectedness is frequently defined as feeling cared for by staff and peers in an academic sense and individually, feeling accepted, respected, included, and supported within the school [27-29].”*

*27.	Libbey HP. Measuring student relationships to school: Attachment, bonding, connectedness, and engagement. The Journal of school health. 2004;74(7):274.*

*28.	Raniti M, Rakesh D, Patton GC, Sawyer SM. The role of school connectedness in the prevention of youth depression and anxiety: a systematic review with youth consultation. BMC public health. 2022;22(1):2152.*

*29.	Pikulski PJ, Pella JE, Casline EP, Hale AE, Drake K, Ginsburg GS. School connectedness and child anxiety. Journal of Psychologists and Counsellors in Schools. 2020;30(1):13-24.*

3. Lines 86-88- are there any other studies to support that statement?

*We have added two additional references.*

*Pg 5, Ln 116: “Research has demonstrated strong negative associations between low school connectedness and concurrent and future self-report symptoms of depression and anxiety [28-30].* *School connectedness has also been found to predict depressive symptoms one year later in girls and boys as well as predict anxiety symptoms one year later in girls [30].”*

*28.	Raniti M, Rakesh D, Patton GC, Sawyer SM. The role of school connectedness in the prevention of youth depression and anxiety: a systematic review with youth consultation. BMC public health. 2022;22(1):2152.*

*29.	Pikulski PJ, Pella JE, Casline EP, Hale AE, Drake K, Ginsburg GS. School connectedness and child anxiety. Journal of Psychologists and Counsellors in Schools. 2020;30(1):13-24.*

*30.	Shochet IM, Dadds MR, Ham D, Montague R. School connectedness is an underemphasized parameter in adolescent mental health: Results of a community prediction study. Journal of Clinical Child & Adolescent Psychology. 2006;35(2):170-9.*

4. It may be beyond the scope of this paper but I would like to read more about "the whole -school mental health intervention" on line 95-96.

*The reference mentioned here is not describing one whole-school intervention, but is rather a review of whole-school mental health focussed interventions. Going into more detail about these is beyond the scope of the paper. However, the authors concluded that there were problems with sustainability and, further, that success partly relied on 'cooperation, training, and involvement of the schools and the young people themselves’. Additionally, they noted that the delivery methods and intervention design were important and needed to appeal to young people, for instance using digital methods.*

*We have expanded on this point slightly with the addition of a large meta analysis of whole-school interventions which found small mental health improvements:*

*Pg 6, Ln 132: “A 2019 meta-analysis which sought to determine the effectiveness of interventions adopting a whole-school approach to enhance young people’s social and emotional development found small, but significant, improvements in participants’ social and emotional adjustment, behavioural adjustment, and internalising symptoms [35].”*

*35.	Goldberg JM, Sklad M, Elfrink TR, Schreurs KM, Bohlmeijer ET, Clarke AM. Effectiveness of interventions adopting a whole school approach to enhancing social and emotional development: a meta-analysis. European Journal of psychology of Education. 2019;34:755-82.*

5. The same comment as #4 for "Health Promoting Schools" on line 97-98.

*Although we agree this is important context for our paper, we are mindful of not adding too much additional information. As such we have added an additional sentence (below), which we feel gives a little more information and links the concept of the whole-school approach and HPS together.*

*Pg 6, Ln 139: “HPS adopt a whole-school approach, where a comprehensive health education curriculum is reinforced by the school's environment and values [37].”*

*37.	Lee A. Health-promoting schools: evidence for a holistic approach to promoting health and improving health literacy. Applied health economics and health policy. 2009;7:11-7.*

6. The aim is clear.

*Thank you.*

7. The methods are clear.

*Thank you.*

8. Lines 160-161- citations 40 and 41- have they been updated?

*We believe these references are the most relevant citations for the statement they support. They were chosen as the first is the most update paper describing the HBSC study and its evolution and the second was chosen because the authors provide details of the measures used.*

9. Tables were all clear to read.

*Thank you.*

10 Discussion Section:

a. Looking at gender identity is important. For the students who identify as neither I would like to know more. It may be beyond the scope of this paper, but in future work it would be interesting to get a sense of identify and for how long someone associated with that identify.

*We agree this would be an interesting additional element. Since the current data do not include this level of detail, we have included this in our future research section, see below.*

*Pg 36, Ln 602:* *“Future research should focus on how to address and improve school connectedness and school environments to improve mental health and mental well-being including potentially, focusing on addressing gender differences in levels of school connectedness. In addition, further details on gender identity could be explored. Future research could also explore mechanisms to reduce the gender gaps in mental health and mental well-being, especially in relation to nonbinary students. Future research could explore the mediating effect of bullying on levels of school connectedness, especially for different gender groups.”*

b. Lines 324-331- in the US there are many high school programs supporting students related to identify- is this the case for your population? You also bring up bulling which would be an important analysis to add in future work.

*In the UK there is no requirement for specific support programmes for students around identity, however, there is a large awareness around this and many schools aim to be supportive and inclusive.*

*Regarding the point about bullying, it is indeed an important element. We were unable to include measures of bullying in this study because of safeguarding concerns raised by the ethics committee. However, we have added this as a consideration for future research.*

*Pg 36, Ln 607: “Future research could explore the mediating effect of bullying on levels of school connectedness, especially for different gender groups.”*

c. Starting line 332- I could assume that the 10s might feel less connected than the 8s as they are thinking about next schooling and possible competition with peers. Is there a more current references than the Kessler reference from 2005? I think you allude to my first comment in (c) with reference 58.

*We have expanded and clarified the explanation given in this section largely by drawing on the paper by Blakemore (2019), which outlines key mechanisms through which adolescents may experience poorer mental health.*

*Pg 32, Ln 501 “With regard to academic year group differences, our data showed for the majority of the mental health, mental well-being, and school connectedness outcomes, Year 10 respondents had poorer outcomes and felt less connected to their school than Year 8 respondents. However in the regression analyses, academic year group was only a significant predictor for poor life satisfaction. According to a large scale meta-analysis conducted in 2021 mental disorders in adulthood started early in the neurodevelopmental phases of life and peaked by mid to late adolescence [71]. Additionally, according to a 2005 study [8], half of all mental health illnesses have manifested by the age of 14. Blakemore (2019), explains mental health illness is more likely to develop in adolescence rather than childhood as a result of neurodevelopmental processes which make the adolescent brain especially vulnerable to stressful or negative environmental events [63]. In addition, adolescence is marked by large increases in oestrogen in girls and testosterone in boys which is implicated in some of the observed mental health disparities found between girls and boys [63]. Thirdly, adolescents experience significant changes in their social environment, such as spending more time with friends and adjusting their self-perception to align with that of their peers [63]. Finally, Blakemore suggests adolescents are more inclined to be risk takers when with peers because of a heightened sensitivity to social exclusion [63]. Given these arguments, it is plausible that Year 8 respondents (aged 12 - 13 years), being in early adolescence, have likely developed fewer mental health challenges compared to Year 10 respondents. Further, Year 10 respondents are faced with increasing school and social pressure as they approach their GCSE exam year, possibly making them more susceptible to mental health difficulties [72].* *Evidence also suggests that school connectedness is related to age, in that older students are less likely to feel connected to their school [29]. This likely explains the differences found in the prevalences of mental health and mental well-being and school connectedness by year group. This is also the probable reason for year group not being an important predictor in the regression analyses in the presence of school connectedness variables such as schoolwork pressure.”*

d. Line 343- can you explain the age/gender issue more clearly?

*We have now expanded on this point to clarify its meaning.*

*Pg 34, Ln 555: “Evidence also suggests that school connectedness is related to age, in that older students are less likely to feel connected to their school [29].”*

e. Line 367- can you explain what you mean by "school inspections?"

*We have now explained this in more detail to make it clearer for an international audience, please see below.*

*Pg 36, Ln 594: “**Schools in England are subject to regular school inspections by the Office for Standards in Education, Children’s Services and Skills (Ofsted). Inspections cover areas such as quality of education, behaviour and attitudes, personal development of students and staff, and leadership and management. Inspections often influence the priorities of the schools, parents, and students [75]* *and provide a mechanism for encouraging schools to implement policies likely to foster greater connectedness and a sense of the school as a community.”*

f. Address if you think your findings can be interpreted beyond England.

*We have added the sentence below which addresses this. We feel our results are largely consistent with what is seen in the national and international literature particularly in relation to gender and poor mental health and the role and importance of socioeconomic status, age, and school connectedness in relation to mental health and well-being. This suggests our proposed points of intervention are relevant to other contexts with similar patterns.*

*Pg 37, Ln 626: “**This study benefits from a large sample of Year 8 and Year10 respondents in South West England, who are a good representation of the students from the 18 schools involved. Our findings are largely consistent with national and international literature, suggesting that the key associations and* *proposed points of intervention are relevant to other contexts with similar patterns—such as rising levels of poor adolescent mental health, gender and socioeconomic disparities, and low levels of school connectedness.”*

Future research clear- possibly some ideas listed above as well.

*Thank you. We have updated the future research section and included your suggestions on gender identity and experiences of bullying.*

*Pg 36, Ln 602: “Future research should focus on how to address and improve school connectedness and school environments to improve mental health and mental well-being including potentially, focusing on addressing gender differences in levels of school connectedness. In addition, further details on gender identity could be explored. Future research could also explore mechanisms to reduce the gender gaps in mental health and mental well-being, especially in relation to nonbinary students. Future research could explore the mediating effect of bullying on levels of school connectedness, especially for different gender groups.”*

Check references. A few are dated. If not seminal, please update. Also check format. For example, reference 24- Journal of School Health (check capitalizations).

*All references have been updated and the formatting of the entire list checked. Below is list of all the references that we have added while updating the paper.*

- *NHS Digital. Mental Health of Children and Young People in England, 2023 - wave 4 follow up to the 2017 survey London2023 [Available from:* [*https://digital.nhs.uk/data-and-information/publications/statistical/mental-health-of-children-and-young-people-in-england/2023-wave-4-follow-up*](https://digital.nhs.uk/data-and-information/publications/statistical/mental-health-of-children-and-young-people-in-england/2023-wave-4-follow-up)*.*
- *Yoon Y, Eisenstadt M, Lereya ST, Deighton J. Gender difference in the change of adolescents’ mental health and subjective wellbeing trajectories. European Child & Adolescent Psychiatry. 2023;32(9):1569-78.*
- *Cela-Bertran X, Peguero G, Serral G, Sánchez-Ledesma E, Martínez-Hernáez A, Pié-Balaguer A. Understanding the relationship between gender and mental health in adolescence: the Gender Adherence Index (GAI). European Child & Adolescent Psychiatry. 2024;33(1):229-40.*
- *Raniti M, Rakesh D, Patton GC, Sawyer SM. The role of school connectedness in the prevention of youth depression and anxiety: a systematic review with youth consultation. BMC public health. 2022;22(1):2152.*
- *Moksnes UK, Løhre A, Lillefjell M, Byrne DG, Haugan G. The Association Between School Stress, Life Satisfaction and Depressive Symptoms in Adolescents: Life Satisfaction as a Potential Mediator. Social Indicators Research. 2016;125(1):339-57.*
- *Cosma A, Stevens G, Martin G, Duinhof EL, Walsh SD, Garcia-Moya I, et al. Cross-National Time Trends in Adolescent Mental Well-Being From 2002 to 2018 and the Explanatory Role of Schoolwork Pressure. Journal of Adolescent Health. 2020;66(6, Supplement):S50-S8.*
- *Goldberg JM, Sklad M, Elfrink TR, Schreurs KM, Bohlmeijer ET, Clarke AM. Effectiveness of interventions adopting a whole school approach to enhancing social and emotional development: a meta-analysis. European Journal of psychology of Education. 2019;34:755-82*
- *Stewart-Brown S, Tennant A, Tennant R, Platt S, Parkinson J, Weich S. Internal construct validity of the Warwick-Edinburgh Mental Well-being Scale (WEMWBS): a Rasch analysis using data from the Scottish Health Education Population Survey. Health and Quality of Life Outcomes. 2009;7(1):15.*
- *Houghton S, Kyron M, Hunter SC, Lawrence D, Hattie J, Carroll A, et al. Adolescents' longitudinal trajectories of mental health and loneliness: The impact of COVID‐19 school closures. Journal of adolescence. 2022;94(2):191-205.*
- *Geulayov G, Mansfield K, Jindra C, Hawton K, Fazel M. Loneliness and self-harm in adolescents during the first national COVID-19 lockdown: results from a survey of 10,000 secondary school pupils in England. Current Psychology. 2024;43(15):14063-74.*
- *Rider GN, McMorris BJ, Gower AL, Coleman E, Eisenberg ME. Health and Care Utilization of Transgender and Gender Nonconforming Youth: A Population-Based Study. Pediatrics. 2018;141(3).*
- *Klinger D, Oehlke S-M, Riedl S, Eschbaum K, Zesch HE, Karwautz A, et al. Mental health of non-binary youth: a systematic review and meta-analysis. Child and Adolescent Psychiatry and Mental Health. 2024;18(1):126.*
- *Moore GF, Cox R, Evans RE, Hallingberg B, Hawkins J, Littlecott HJ, et al. School, peer and family relationships and adolescent substance use, subjective wellbeing and mental health symptoms in Wales: A cross sectional study. Child indicators research. 2018;11:1951-65.*
- *Solmi M, Radua J, Olivola M, Croce E, Soardo L, Salazar de Pablo G, et al. Age at onset of mental disorders worldwide: large-scale meta-analysis of 192 epidemiological studies. Molecular psychiatry. 2022;27(1):281-95.*

Overall, very well done.

I look forward to reading more in this area.

Keep up the good work and consider partnerships beyond your local area of England.

**Reviewer #2:**

This study examined the mental health outcomes by gender, level of school connectedness, and socioeconomic status among 5193 students in the UK. The findings highlighted the needs for mental health interventions and discrimination prevention for high-risk groups among this population. The practical and research implications of the findings are significant. The authors did a very good job providing a rationale for this study. The results were clearly laid out and well organized. However, I point their attention to a few issues that should be addressed.

1. Given the noted gender differences in the mental health outcomes and school related outcomes, the authors might consider presenting all results separately by gender. At least for Table 1 and Table 2, it would be nice to have the statistics by gender. This would better characterize the subgroups.

*We have updated Table 1 (Pg 13&14) to include a gender breakdown for these different demographic characteristics. The data in Table 2 is already available by gender, this is given in Table 3 (Pg 18&19).*

2. You have a lot of results. It would be nice to include some data visualization in your paper. This would help the readers digest the findings and tell a better story.

*We have added forest plots of the regression analyses (suggested below), as a supplementary file, which we feel support the reader in interpreting the results.*

3. One major concern is the lack of inferential statistical analysis. A regression model would help better understand how gender, school connectedness, and socioeconomic status were linked to mental health outcomes, and also how these factors might interplay with each other. Better understanding of the pathway would guide interventions. The study sample size is decent, which means enough power for regression analysis.

*We have since conducted regression analyses of all the mental health and mental well-being outcomes (life satisfaction, depression, anxiety, and loneliness), using gender, academic year, socioeconomic status and measures of school connectedness as predictors. Key results are discussed on Pg 27, with results presented in Table 6 on pages 29 & 30. As a result of this change we have updated the entire paper including the introduction, aim, methods, results and discussion. Our regression results have confirmed the overall messages of the paper – gender and school connectedness are strongly associated with poor mental health, with girls consistently reporting poorer mental health outcomes than boys, and school connectedness showing strong associations with poor mental health outcomes.*

4. The handling of missing data: did the authors conduct comparison analysis to look at those excluded due to missing > 20% data to those included in the analysis sample? This is important.

*The 20% mentioned above is referring to how the data within the scale based questions were handled, not the actual levels of missing data. We have reworded the section on how missing data were handled in the methods sections (see below). We have also added the levels of missing data to Table 1 and 2 and we have added a sentence on the levels of missing data on Pg13 (see below).*

*Pg 11 Ln 250: “**For all scale-based questions (symptoms of depression, anxiety, and mental well-being), a 20% missing data threshold was used, meaning if a student did not respond to 20% or more of the scale’s items, their total score was not calculated and their response was counted as missing. However, if a student missed less than 20% of the scale items, their average response to the answered items was imputed in the missing item’s place, resulting in a complete scale score. For the loneliness, classmate support, teacher support, and peer support scales if a student did not answer one of the scale questions, their final score was not calculated and their response was counted as missing.”*

*Pg13 ln 297: “**Over 80% (4326) of respondents provided complete responses. Missing data across all the different variables were relatively low, <10% for most, with some variables (family structure, peer support and teacher support) having slightly higher missing rates, but all were <17%.”*

5. What was the response rate of the survey/study? Is the study sample representative of the overall student population? This should be specified in the Methods section and potentially discussed as a limitation, if appropriate.

*We have added the overall response rate. School response rates were discussed in the first paragraph of the results section where we detail the school characteristics. However, we have now moved some of this to the methods sections.*

*Pg 8 ln 185: “In total, 18 schools completed the survey across the two academic years (2020/2021 and 2021/2022), with six schools taking part during the 2020/2021 academic year. Students in two of these six schools completed the survey whilst locked down at home during the Covid-19 pandemic. Response rates ranged from 16% to 97%, with an overall response rate of 74%. The two schools that completed the survey whilst at home had the lowest response rates.”*
