# Supplementary material for: Prevalence of mental health, mental well-being, and school connectedness in UK secondary school students in 2021/2022: Results from the South West - School Health Research Network pilot study
Source: PLoS One. 2026 Aug 28;21(8):e0355874. doi: 10.1371/journal.pone.0355874 (PMC13524283; doi:10.1371/journal.pone.0355874)
Supplement: Supplementary file 9 [file pone.0355874.s009.docx]

**Response to reviewers**

Manuscript Title: Prevalence of mental health, mental well-being, and school connectedness in UK secondary school students in 2021/2022: results from the South West - School Health Research Network pilot study. Manuscript Number: PONE-D-25-55322

We thank the reviewers for these helpful minor suggestions and have addressed these as follows:

The authors have acknowledged the context of the Covid pandemic as recommended, but the inclusion of the sentence ‘However, all data collection took place in the aftermath of pandemic-related disruption’ (p8, 178) seems confusing, given the earlier statement that ‘Students in two of these six schools completed the survey remotely during home learning as a result of the Covid-19 pandemic restrictions and school closures’ (p8, 174-176). I suggest deleting the sentence on line 178 beginning’ However, all data collection…’

We have deleted the sentence as suggested, however, we felt it was still important to acknowledge that these remaining schools completed the survey during the aftermath of the pandemic. We have therefore updated the previous sentence, see below.

**Pg 7, Ln 161**: “All remaining participating schools completed the survey after restrictions had eased and students had returned to in-person schooling, although schools continued to be influenced by the aftermath of the COVID-19 pandemic.”

The sentence: ‘ However, if the broader context of the pandemic schools varied in the methods and extent of contact with students during school closures.’ (P8, 181-2) should be rewritten as: ‘However, in the broader context of the pandemic, schools varied in the methods and extent of contact with students during school closures.’

We have corrected the sentence as suggested (**Pg 8, 165**).
