# Supplementary material for: Prevalence of mental health, mental well-being, and school connectedness in UK secondary school students in 2021/2022: Results from the South West - School Health Research Network pilot study
Source: PLoS One. 2026 Aug 28;21(8):e0355874. doi: 10.1371/journal.pone.0355874 (PMC13524283; doi:10.1371/journal.pone.0355874)
Supplement: Peer review history

# Author comment: Author response to Decision Letter 0

**DOI:** 10.1371/journal.pone.0355874.r001

13 Oct 2025

Attachment Submitted filename: Response to reviewers_10102025.docx

# Peer review: Decision Letter 0

**DOI:** 10.1371/journal.pone.0355874.r002

**Authors:** Javier Fagundo-Rivera

8 Mar 2026

Dear Dr. Albers,

Thank you for submitting your manuscript to PLOS ONE. After careful consideration, we feel that it has merit but does not fully meet PLOS ONE’s publication criteria as it currently stands. Therefore, we invite you to submit a revised version of the manuscript that addresses the points raised during the review process.

Please submit your revised manuscript by Apr 22 2026 11:59PM. If you will need more time than this to complete your revisions, please reply to this message or contact the journal office at plosone@plos.org. When you're ready to submit your revision, log on to https://www.editorialmanager.com/pone/ and select the 'Submissions Needing Revision' folder to locate your manuscript file.

- A letter that responds to each point raised by the academic editor and reviewer(s). You should upload this letter as a separate file labeled 'Response to Reviewers'.
- A marked-up copy of your manuscript that highlights changes made to the original version. You should upload this as a separate file labeled 'Revised Manuscript with Track Changes'.
- An unmarked version of your revised paper without tracked changes. You should upload this as a separate file labeled 'Manuscript'.

If you would like to make changes to your financial disclosure, please include your updated statement in your cover letter. Guidelines for resubmitting your figure files are available below the reviewer comments at the end of this letter.

If applicable, we recommend that you deposit your laboratory protocols in protocols.io to enhance the reproducibility of your results. Protocols.io assigns your protocol its own identifier (DOI) so that it can be cited independently in the future. For instructions see: https://journals.plos.org/plosone/s/submission-guidelines#loc-laboratory-protocols. Additionally, PLOS ONE offers an option for publishing peer-reviewed Lab Protocol articles, which describe protocols hosted on protocols.io. Read more information on sharing protocols at https://plos.org/protocols?utm_medium=editorial-email&utm_source=authorletters&utm_campaign=protocols.

We look forward to receiving your revised manuscript.

Kind regards,

Javier Fagundo-Rivera, PhD

Academic Editor

PLOS One

Journal Requirements:

When submitting your revision, we need you to address these additional requirements.

1.Please ensure that your manuscript meets PLOS ONE's style requirements, including those for file naming. The PLOS ONE style templates can be found at

https://journals.plos.org/plosone/s/file?id=wjVg/PLOSOne_formatting_sample_main_body.pdf and

https://journals.plos.org/plosone/s/file?id=ba62/PLOSOne_formatting_sample_title_authors_affiliations.pdf

2. Please note that funding information should not appear in any section or other areas of your manuscript. We will only publish funding information present in the Funding Statement section of the online submission form. Please remove any funding-related text from the manuscript.

3. Thank you for stating the following financial disclosure:

“This study was funded by the National Institute for Health and Care Research (NIHR) School for Public Health Research (SPHR), Grant Reference Number PD-SPH-2015. The views expressed are those of the author(s) and not necessarily those of the NIHR or the Department of Health and Social Care. EK is supported by an NIHR Senior Investigator Award and is a co-applicant of the NIHR School of Public Health Research (Fuse) and Director of the NIHR Applied Research Collaboration North East and North Cumbria.”

Please state what role the funders took in the study.  If the funders had no role, please state: "The funders had no role in study design, data collection and analysis, decision to publish, or preparation of the manuscript."

If this statement is not correct you must amend it as needed.

Please include this amended Role of Funder statement in your cover letter; we will change the online submission form on your behalf.

4. Thank you for uploading your study's underlying data set. Unfortunately, the repository you have noted in your Data Availability statement does not qualify as an acceptable data repository according to PLOS's standards.

At this time, please upload the minimal data set necessary to replicate your study's findings to a stable, public repository (such as figshare or Dryad) and provide us with the relevant URLs, DOIs, or accession numbers that may be used to access these data. For a list of recommended repositories and additional information on PLOS standards for data deposition, please see https://journals.plos.org/plosone/s/recommended-repositories.

5. In the online submission form, you indicated that “The dataset analysed during the current study will be available from the University of Bristol data archive, https://data.bris.ac.uk/data/. This dataset has a controlled access level, which means it is not available via direct download but must be requested.”

All PLOS journals now require all data underlying the findings described in their manuscript to be freely available to other researchers, either 1. In a public repository, 2. Within the manuscript itself, or 3. Uploaded as supplementary information.

This policy applies to all data except where public deposition would breach compliance with the protocol approved by your research ethics board. If your data cannot be made publicly available for ethical or legal reasons (e.g., public availability would compromise patient privacy), please explain your reasons on resubmission and your exemption request will be escalated for approval.

6. Please include a separate caption for each figure in your manuscript.

7.  Please include captions for your Supporting Information files at the end of your manuscript, and update any in-text citations to match accordingly. Please see our Supporting Information guidelines for more information: http://journals.plos.org/plosone/s/supporting-information.

8. If the reviewer comments include a recommendation to cite specific previously published works, please review and evaluate these publications to determine whether they are relevant and should be cited. There is no requirement to cite these works unless the editor has indicated otherwise.

**Additional Editor Comments:**

Dear Authors,

Your manuscript has now been evaluated by three reviewers. Based on their comments and recommendations, the editorial decision is Major Revision.

We kindly ask you to submit a revised version of your manuscript addressing all the comments raised by the reviewers. Please also include a detailed response letter in which you reply to each reviewer comment, clearly indicating the changes made in the manuscript and providing any relevant explanations.

We look forward to receiving your revised manuscript.

Best regards,

[Note: HTML markup is below. Please do not edit.]

Reviewer's Responses to Questions

**Comments to the Author**

1. Is the manuscript technically sound, and do the data support the conclusions?

Reviewer #1: Yes

Reviewer #2: Yes

Reviewer #3: Partly

**********

2. Has the statistical analysis been performed appropriately and rigorously?

Reviewer #1: Yes

Reviewer #2: Yes

Reviewer #3: Yes

**********

3. Have the authors made all data underlying the findings in their manuscript fully available?

The PLOS Data policy

Reviewer #1: Yes

Reviewer #2: No

Reviewer #3: Yes

**********

4. Is the manuscript presented in an intelligible fashion and written in standard English?

Reviewer #1: Yes

Reviewer #2: Yes

Reviewer #3: Yes

**********

**Reviewer #1:**

Manuscript Title: Prevalence of mental health, mental well-being, and school connectedness in UK secondary school students: results from the South West - School Health Research Network pilot study.

Manuscript Number: PONE-D-25-55322

Overall Comments: The manuscript is well-written and logically organized. However, addressing the following minor points will strengthen the clarity and rigor of the work.

Specific Comments:

Abstract: Please include key findings from the regression analysis, providing precise estimates (e.g., AOR with CI) alongside the existing descriptive statistics.

Methods:

Data Collection Rationale: Provide a brief justification (2–3 lines) for the use of self-reported data.

Data Quality & Non-Response: Detail how data quality was maintained, specifically addressing non-response rates and the characteristics of those who did not participate.

Participant Understanding: Explain how you ensured respondents understood the questions in the self-administered format. Was there a mechanism for participants to contact the research team for clarification or further queries?

Variables: Clearly define the primary explanatory variables and the covariates included in the regression models.

Discussion:

Limitations: Expand on the study's limitations, particularly those inherent to cross-sectional designs, and discuss how these could be addressed or mitigated in future research.

Policy Implications: Introduce a dedicated subsection discussing the policy implications derived directly from your findings.

**Reviewer #2:**

This is a really interesting topic and the efforts behind making it work are really obvious. I would recommend the paper for publication. However, I have minor details to be addressed.

Title: The title should contain the period of the study (2021-2022)

Abstract: Tools should be written in the Methods section (e.g.). GAD-7 for anxiety, SMFQ for depression, and so on for the rest of the tools, in addition to the method of data collection. To accommodate the word limit, try minimising the size of the results and conclusion subsections while keeping the concept. (DELETE THE WORD COUNT FROM YOUR MANUSCRIPT)

Background: Well-written and very informative

Methods: Why were only the students of Years 8 and 10 chosen? Why not 9 for example?

Please provide an explanation. Please cite the IMD in line 169.

Switch the Analysis and Ethics subsections and minimise the Analysis subsection.

Results: Explain the process of weighting your variables if possible. Fix the 40% in line 269

It would be of great value if ethnicity were involved in the analysis as it could have significant results.

Discussion: Excellent

Conclusion: Instead of "we have shown", "this paper clearly demonstrates" is more acceptable.

**Reviewer #3:**

I didn’t review the first submission of this paper so I haven’t commented in any detail on whether the original reviewers’ recommendations have been fully met. This is an interesting study with a large sample exploring associations between adolescents’ feelings of school connectedness and their mental health. The findings are clearly reported and the findings on sexual identity are particularly useful. However, there needs to be much more emphasis on the context of the study, especially given that school connectedness is a major theme. The study took place during the global pandemic and some children completed the survey while schools were closed. This is highly likely to have impacted on experience and perceptions of school connectedness and on their mental health directly. More consideration of differences between schools, especially in relation to perceptions of connectedness, would also be valuable. As it stands, the paper is very focused on differences in the student population but a ‘whole school approach’ acknowledges that diversity between schools can shape student experience and mental health.

Methods

It would be worth including some justification for the choice of measures in the main body of the paper. For instance, there are other more calibrated tools available for measuring school climate or environment such as Cornell’s Authoritative School Climate Survey (ASCS). You need to confirm in the main body of the paper that all measures used had been validated for use with adolescents.

Timing of survey – we are told that two schools completed the survey during lockdown due to the pandemic. Did students in the other participating schools complete the survey following lockdown? More clarity is needed regarding how school closures in England affected both survey completion and the children participating in this study. The section on School Characteristics should include information on how schools included in this study maintained contact with students during the pandemic.

Results

It would be helpful to know whether significant variations (particularly in respect of school connectedness) were found between schools – if so, can these be attributed to how they experienced or responded to the pandemic or to the size of school?

Discussion

Generally, much more needs to be said about the context of the pandemic in the limitations section – this was an extraordinary period and school closures are highly likely to have impacted on both the mental health of the children participating in this study and on their perceptions of school connectedness. The researchers were unfortunate in the timing of their study and they have my sympathy but this issue is almost completely ignored in the paper.

**********

what does this mean?). If published, this will include your full peer review and any attached files.

If you choose “no”, your identity will remain anonymous but your review may still be made public.

**Do you want your identity to be public for this peer review?**  For information about this choice, including consent withdrawal, please see our Privacy Policy

Reviewer #1: No

Reviewer #2: **Yes:**  Weaam Abdallah

Reviewer #3: No

**********

[NOTE: If reviewer comments were submitted as an attachment file, they will be attached to this email and accessible via the submission site. Please log into your account, locate the manuscript record, and check for the action link "View Attachments". If this link does not appear, there are no attachment files.]

To ensure your figures meet our technical requirements, please review our figure guidelines: https://journals.plos.org/plosone/s/figures

You may also use PLOS’s free figure tool, NAAS, to help you prepare publication quality figures: https://journals.plos.org/plosone/s/figures#loc-tools-for-figure-preparation.

NAAS will assess whether your figures meet our technical requirements by comparing each figure against our figure specifications.

Attachment Submitted filename: Reviewers Comments.docx

# Author comment: Author response to Decision Letter 1

**DOI:** 10.1371/journal.pone.0355874.r003

18 Jun 2026

Response to reviewers

Manuscript Title: Prevalence of mental health, mental well-being, and school connectedness in UK secondary school students in 2021/2022: results from the South West - School Health Research Network pilot study. Manuscript Number: PONE-D-25-55322

We thank all three reviewers for these helpful suggestions, all of which have strengthened our manuscript. We note that several of the methodological details are described more extensively in the published SW-SHRN protocol paper (Creation of a Pilot School Health Research Network in an English Education Infrastructure to Improve Adolescent Health and Well-Being: A Study Protocol), including the survey procedures, use of self-report measures, co-production processes, and the wider implementation context during the COVID-19 pandemic. However, we have incorporated additional details into the revised manuscript. The protocol paper is referenced at the beginning of the methods section (Pg 6, Ln134).

Reviewer #1:

Overall Comments: The manuscript is well-written and logically organized. However, addressing the following minor points will strengthen the clarity and rigor of the work.

Specific Comments:

Abstract:

Please include key findings from the regression analysis, providing precise estimates (e.g., AOR with CI) alongside the existing descriptive statistics.

Key findings from the regression analysis have now been added, and the abstract has been revised to meet the maximum word limit. See Pg 2, Ln 29

Methods:

Data Collection Rationale: Provide a brief justification (2–3 lines) for the use of self-reported data.

We have added a brief justification for the use of self-reported data within the “Methods” section (see Pg 6, Ln 146). Self-report surveys are widely regarded as best practice in adolescent school-based health research because they enable the efficient collection of large-scale data on health behaviours, well-being, social relationships, and school experiences that are not readily observable through administrative or clinical records. Self-report methods are also central to established national and international adolescent health surveys including the Health Behaviour in School-aged Children (HBSC) study and the wider School Health Research Network (SHRN) infrastructure. Confidential self-report approaches have been shown to improve disclosure of sensitive behaviours and experiences among adolescents, thereby supporting longitudinal monitoring of health and well-being over time.

Data Quality & Non-Response: Detail how data quality was maintained, specifically addressing non-response rates and the characteristics of those who did not participate.

In total, 6868 student names were provided by the schools, of which 5193 (76%) completed the survey. We have no data regarding the 24% that did not complete the survey so cannot explore the characteristics of this group. However, of those that did complete the survey over 80% provided complete responses.

Tables 1 and 2 include the level of missing data across the different outcome measures. As explained on Pg 13, Ln 306 (Sample characteristics section). Missingness was relatively low with most questions having <10% missing. However, three measures; student, teacher, and peer support; were missing for approximately 10%. In the regression analysis we used complete-case analysis. However, we have added an additional supplementary file (S3) which shows the frequencies and proportions across measures for these three variables for students with and without data. From these data it is evident that the distribution across these variables was comparable between those with and without data. However, there is some indication that participants with missing student support data had slightly poorer outcome measures, suggesting missingness may not have been completely random. We have added a description of this within the ‘Sample characteristics’ section, see Pg 13, Ln 310.

Participant Understanding: Explain how you ensured respondents understood the questions in the self-administered format. Was there a mechanism for participants to contact the research team for clarification or further queries?

Prior to survey implementation, we worked closely with a Young Persons’ Advisory Group to review the survey content, wording, and accessibility of questions to ensure that items were understandable and age-appropriate for adolescents. Feedback from young people informed adaptations to language and phrasing throughout the survey. In addition, for several standardised questionnaire items, supplementary explanations or simplified wording were provided for terms that adolescents commonly queried during pilot discussions or previous survey administrations. During survey completion, participants were also able to seek clarification from members of the research team or school staff present during administration if they were unsure about the meaning of particular questions or terms. We have clarified this process within the manuscript, see Pg 7, Ln151.

Variables: Clearly define the primary explanatory variables and the covariates included in the regression models.

We have added these variables for clarity (Pg 11, Ln 258) and further details of the measures is provided on Pg 12, Ln 275.

Discussion:

Limitations: Expand on the study's limitations, particularly those inherent to cross-sectional designs, and discuss how these could be addressed or mitigated in future research.

In the “Strengths and limitations” section we have added two sentences addressing this important reminder that our data are cross-sectional (see Pg 36, Ln 617). Other limitations such as missingness in our data, the pandemic context, and improving how to capture gender identity are also discussed.

Policy Implications: Introduce a dedicated subsection discussing the policy implications derived directly from your findings.

We have reworked part of the discussion section (see Pg 34, Ln 564) highlighting key policy implication and further added to these. These appear in a specific section; “Policy implications”, where we explicitly discuss the need for schools to recognise gender differences in experiences of mental health and school connectedness. We also discuss the importance of school connectedness and how schools should prioritise whole school and health promoting approaches and the role that Ofsted could play in that.

Reviewer #2:

This is a really interesting topic and the efforts behind making it work are really obvious. I would recommend the paper for publication. However, I have minor details to be addressed.

Title: The title should contain the period of the study (2021-2022)

Title has been amended: “Prevalence of mental health, mental well-being, and school connectedness in UK secondary school students in 2021/2022: results from the South West - School Health Research Network pilot study.”

Abstract:

Tools should be written in the Methods section (e.g.). GAD-7 for anxiety, SMFQ for depression, and so on for the rest of the tools, in addition to the method of data collection.

Tool names have been added (see Pg 2, Ln 38).

To accommodate the word limit, try minimising the size of the results and conclusion subsections while keeping the concept.

We have reworked the abstract to include the above as well as additions suggested by Reviewer 1. The abstract is in keeping with the word limit.

(DELETE THE WORD COUNT FROM YOUR MANUSCRIPT)

Deleted

Background: Well-written and very informative

Methods:

Why were only the students of Years 8 and 10 chosen? Why not 9 for example? Please provide an explanation.

Students in Years 8 and 10 were selected to align with the sampling strategy used within the wider School Health Research Network (SHRN) model and to capture two distinct developmental stages during secondary education: early adolescence (Year 8; aged 12–13 years) and mid-adolescence (Year 10; aged 14–15 years). Year 7 were not included because this represents a key transitional year from primary to secondary school and older year groups were not included as these include key examination periods (e.g. Year 11, 12 and 13) so we avoided these year groups to ensure we did not increase burden on students and schools. Selecting Years 8 and 10 therefore enabled collection of data from students who were more established within the school environment while minimising academic disruption.

This approach also reduced participant burden on schools while still enabling meaningful comparisons across age groups and developmental transitions. Including all year groups was not considered feasible during the pilot implementation phase, particularly given the operational pressures schools face. Years 8 and 10 were therefore selected as pragmatic cohorts that could provide representation across lower and upper secondary school experiences.

We have added a brief explanation within the “Inclusion and exclusion criteria of schools and students” section, see Pg 7, Ln 167.

Please cite the IMD in line 169.

Citation included (Pg 9, Ln 203).

Switch the Analysis and Ethics subsections and minimise the Analysis subsection.

We have switched the analyses and ethics subsections around (Pg 10, 237).

We have reduced the analysis section, specifically removing the section regarding the assumptions of OLR and reduced the model comparison element. These details have been moved to S2 (Pg 11, 258).

Results: Explain the process of weighting your variables if possible.

We applied survey weights to account for differential response rates across schools. Weighting was implemented using the ‘survey’ package in R. We have reworded that section to make this clearer (see Pg 10, Ln 239).

Fix the 40% in line 269

Erroneous “J” deleted (Pg 15, Ln 330).

It would be of great value if ethnicity were involved in the analysis as it could have significant results.

We completely agree ethnicity is an important aspect within this broader picture of mental health and well-being and school connectedness. Although ethnicity data were collected and are reported descriptively, we did not include it in the main analyses because the relatively small proportion of participants from minority ethnic backgrounds (~14% other than white) limited the feasibility of conducting robust ethnicity-based analyses. Investigating ethnicity and its intersection with other demographic factors would require more targeted analyses and was therefore considered beyond the scope of the present study.

Discussion: Excellent

Conclusion: Instead of "we have shown", "this paper clearly demonstrates" is more acceptable.

Opening line of the conclusion has been amended.

Reviewer #3:

I didn’t review the first submission of this paper so I haven’t commented in any detail on whether the original reviewers’ recommendations have been fully met.

This is an interesting study with a large sample exploring associations between adolescents’ feelings of school connectedness and their mental health. The findings are clearly reported and the findings on sexual identity are particularly useful.

However, there needs to be much more emphasis on the context of the study, especially given that school connectedness is a major theme. The study took place during the global pandemic and some children completed the survey while schools were closed. This is highly likely to have impacted on experience and perceptions of school connectedness and on their mental health directly.

We have added a section, at the start of the discussion section, called “Covid-19 pandemic context”. In this section we discuss this context more fully, please see Pg 30, Ln 449.

More consideration of differences between schools, especially in relation to perceptions of connectedness, would also be valuable. As it stands, the paper is very focused on differences in the student population but a ‘whole school approach’ acknowledges that diversity between schools can shape student experience and mental health.

Our analyses focused on individual-level associations between school connectedness and mental health and well-being among young people rather than on variation between schools. As participants were nested within schools, school-level clustering was accounted for statistically to address the non-independence of observations and produce more robust standard errors. However, the study was not designed to investigate school-level effects or contextual differences between schools, and schools were therefore treated primarily as clustering units rather than substantive units of comparison.

That said we do feel this is important observation and we have therefore addressed this in the manuscript (Pg 8, Ln 190) as well as provided a supplementary table (S1) with the range of proportions across schools as a descriptive comparison of the variation across schools.

Methods:

It would be worth including some justification for the choice of measures in the main body of the paper. For instance, there are other more calibrated tools available for measuring school climate or environment such as Cornell’s Authoritative School Climate Survey (ASCS). You need to confirm in the main body of the paper that all measures used had been validated for use with adolescents.

The measures included in the student survey were selected because they had previously been used within the established HBSC and Welsh SHRN survey which we modelled SW-SHRN on and had demonstrated acceptability and feasibility for administration within school settings. The survey drew upon validated adolescent health and well-being instruments widely used in school-based population research, including measures relating to school connectedness, mental health and well-being, risk behaviours, peer relationships, and social media use. We will clarify in the revised manuscript that all measures included had either previously demonstrated validity and reliability in adolescent populations or were adapted from established national and international adolescent health surveys.

We acknowledge that there are a wide range of measures surrounding school environment and school climate, however, survey length and feasibility were important considerations given the need to administer the questionnaire during a single school lesson alongside a broad range of health and well-being domains. Consequently, brief validated measures that had already been successfully implemented within existing school health research networks/surveys were prioritised.

We have reworded the measures section in the paper to make this clearer, see Pg 9, Ln 196.

Timing of survey – we are told that two schools completed the survey during lockdown due to the pandemic. Did students in the other participating schools complete the survey following lockdown?

We have clarified this point in the manuscript, see Pg 8, Ln 174. Two participating schools completed the survey during periods of COVID-19 restrictions and partial school closure, meaning students completed the survey remotely during home learning. The remaining participating schools completed the survey after national lockdown restrictions had eased and students had returned to in-person schooling. However, all participating schools were still operating within the broader context of pandemic-related disruption.

More clarity is needed regarding how school closures in England affected both survey completion and the children participating in this study.

We have added a section (titled Covid-19 pandemic context, Pg 30, Ln 449), to the beginning of the discussion section, where we specifically address this. We acknowledge the impact this had on the research, and we recognise this as an important contextual factor when interpreting our findings.

The section on School Characteristics should include information on how schools included in this study maintained contact with students during the pandemic.

Participating schools maintained contact with students during periods of school closure using a range of approaches consistent with national guidance at the time, including online learning platforms, virtual lessons and email communication. In schools where students were learning remotely, surveys were administered through these existing infrastructures (e.g. the survey link was emailed out for students to complete online in their own time at hom

Attachment Submitted filename: Response to Reviewers.docx

# Peer review: Decision Letter 1

**DOI:** 10.1371/journal.pone.0355874.r004

**Authors:** Javier Fagundo-Rivera

14 Jul 2026

Dear Dr. Albers,

Thank you for submitting your manuscript to PLOS ONE. After careful consideration, we feel that it has merit but does not fully meet PLOS ONE’s publication criteria as it currently stands. Therefore, we invite you to submit a revised version of the manuscript that addresses the points raised during the review process.

==============================

**ACADEMIC EDITOR:**

Dear Authors,

Thank you for submitting your manuscript entitled “ Prevalence of mental health, mental well-being, and school connectedness in UK secondary school students in 2021/2022: results from the South West - School Health Research Network pilot study. ” (Manuscript Number: PONE-D-25-55322R1) to Plos One.

After evaluation by three independent reviewers and editorial assessment, the current decision is Minor Revision.

The reviewers recognize the relevance, originality, and potential contribution of this work. Overall, the manuscript is well prepared and addresses an important topic. However, several points were identified that would benefit from clarification and minor improvement before the manuscript can be considered for publication.

Please revise the manuscript carefully by addressing each reviewer comment point-by-point in a detailed response letter. We encourage you to clearly indicate all modifications made in the revised manuscript, preferably using track changes or highlighted text.

We will accept this publication after these minor revisions are considered by the authors.

We look forward to receiving your revised submission and appreciate your contribution to the field.

Sincerely,

Javier Fagundo-Rivera, PhD

Academic Editor

==============================

Please submit your revised manuscript by Aug 28 2026 11:59PM. If you will need more time than this to complete your revisions, please reply to this message or contact the journal office at plosone@plos.org. When you're ready to submit your revision, log on to https://www.editorialmanager.com/pone/ and select the 'Submissions Needing Revision' folder to locate your manuscript file.

- A letter that responds to each point raised by the academic editor and reviewer(s). You should upload this letter as a separate file labeled 'Response to Reviewers'.
- A marked-up copy of your manuscript that highlights changes made to the original version. You should upload this as a separate file labeled 'Revised Manuscript with Track Changes'.
- An unmarked version of your revised paper without tracked changes. You should upload this as a separate file labeled 'Manuscript'.

If you would like to make changes to your financial disclosure, please include your updated statement in your cover letter. Guidelines for resubmitting your figure files are available below the reviewer comments at the end of this letter.

If applicable, we recommend that you deposit your laboratory protocols in protocols.io to enhance the reproducibility of your results. Protocols.io assigns your protocol its own identifier (DOI) so that it can be cited independently in the future. For instructions see: https://journals.plos.org/plosone/s/submission-guidelines#loc-laboratory-protocols. Additionally, PLOS ONE offers an option for publishing peer-reviewed Lab Protocol articles, which describe protocols hosted on protocols.io. Read more information on sharing protocols at https://plos.org/protocols?utm_medium=editorial-email&utm_source=authorletters&utm_campaign=protocols.

As the corresponding author, your ORCID iD is verified in the submission system and will appear in the published article. PLOS supports the use of ORCID, and we encourage all coauthors to register for an ORCID iD and use it as well. Please encourage your coauthors to verify their ORCID iD within the submission system before final acceptance, as unverified ORCID iDs will not appear in the published article. *Only*  the individual author can complete the verification step; PLOS staff *cannot*  verify ORCID iDs on behalf of authors.

We look forward to receiving your revised manuscript.

Kind regards,

Javier Fagundo-Rivera, PhD

Academic Editor

PLOS One

Journal Requirements:

If the reviewer comments include a recommendation to cite specific previously published works, please review and evaluate these publications to determine whether they are relevant and should be cited. There is no requirement to cite these works unless the editor has indicated otherwise.

Please review your reference list to ensure that it is complete and correct. If you have cited papers that have been retracted, please include the rationale for doing so in the manuscript text, or remove these references and replace them with relevant current references. Any changes to the reference list should be mentioned in the rebuttal letter that accompanies your revised manuscript. If you need to cite a retracted article, indicate the article’s retracted status in the References list and also include a citation and full reference for the retraction notice.

**Additional Editor Comments:**

Dear Authors,

Thank you for submitting your manuscript entitled “ Prevalence of mental health, mental well-being, and school connectedness in UK secondary school students in 2021/2022: results from the South West - School Health Research Network pilot study. ” (Manuscript Number: PONE-D-25-55322R1) to Plos One.

After evaluation by three independent reviewers and editorial assessment, the current decision is Minor Revision.

The reviewers recognize the relevance, originality, and potential contribution of this work. Overall, the manuscript is well prepared and addresses an important topic. However, several points were identified that would benefit from clarification and minor improvement before the manuscript can be considered for publication.

Please revise the manuscript carefully by addressing each reviewer comment point-by-point in a detailed response letter. We encourage you to clearly indicate all modifications made in the revised manuscript, preferably using track changes or highlighted text.

We will accept this publication after these minor revisions are considered by the authors.

We look forward to receiving your revised submission and appreciate your contribution to the field.

Sincerely,

Javier Fagundo-Rivera, PhD

Academic Editor

[Note: HTML markup is below. Please do not edit.]

Reviewers' comments:

Reviewer's Responses to Questions

**Comments to the Author**

Reviewer #1: All comments have been addressed

Reviewer #3: (No Response)

**********

2. Is the manuscript technically sound, and do the data support the conclusions?

Reviewer #1: Yes

Reviewer #3: Yes

**********

3. Has the statistical analysis been performed appropriately and rigorously?

Reviewer #1: Yes

Reviewer #3: Yes

**********

4. Have the authors made all data underlying the findings in their manuscript fully available?

The PLOS Data policy

Reviewer #1: No

Reviewer #3: Yes

**********

5. Is the manuscript presented in an intelligible fashion and written in standard English?

Reviewer #1: Yes

Reviewer #3: Yes

**********

**Reviewer #1:**

Thank you for the revision and resubmission of this manuscript. All previous comments have been thoroughly addressed by the authors. I have no further comments.

**Reviewer #3:**

The authors have responded satisfactorily to the reviewers’ comments.

A couple of minor issues are outstanding:

The authors have acknowledged the context of the Covid pandemic as recommended, but the inclusion of the sentence ‘However, all data collection took place in the aftermath of pandemic-related disruption’ (p8, 178) seems confusing, given the earlier statement that ‘Students in two of these six schools completed the survey remotely during home learning as a result of the Covid-19 pandemic restrictions and school closures’ (p8, 174-176). I suggest deleting the sentence on line 178 beginning’ However, all data collection…’

The sentence: ‘ However, if the broader context of the pandemic schools varied in the methods and extent of contact with students during school closures.’ (P8, 181-2) should be rewritten as: ‘However, in the broader context of the pandemic, schools varied in the methods and extent of contact with students during school closures.’

**********

what does this mean?). If published, this will include your full peer review and any attached files.

If you choose “no”, your identity will remain anonymous but your review may still be made public.

**Do you want your identity to be public for this peer review?**  For information about this choice, including consent withdrawal, please see our Privacy Policy

Reviewer #1: No

Reviewer #3: No

**********

[NOTE: If reviewer comments were submitted as an attachment file, they will be attached to this email and accessible via the submission site. Please log into your account, locate the manuscript record, and check for the action link "View Attachments". If this link does not appear, there are no attachment files.]

To ensure your figures meet our technical requirements, please review our figure guidelines: https://journals.plos.org/plosone/s/figures

You may also use PLOS’s free figure tool, NAAS, to help you prepare publication quality figures: https://journals.plos.org/plosone/s/figures#loc-tools-for-figure-preparation.

NAAS will assess whether your figures meet our technical requirements by comparing each figure against our figure specifications.

# Author comment: Author response to Decision Letter 2

**DOI:** 10.1371/journal.pone.0355874.r005

21 Jul 2026

We thank the reviewers for these helpful minor suggestions and have addressed these as follows:

The authors have acknowledged the context of the Covid pandemic as recommended, but the inclusion of the sentence ‘However, all data collection took place in the aftermath of pandemic-related disruption’ (p8, 178) seems confusing, given the earlier statement that ‘Students in two of these six schools completed the survey remotely during home learning as a result of the Covid-19 pandemic restrictions and school closures’ (p8, 174-176). I suggest deleting the sentence on line 178 beginning’ However, all data collection…’

We have deleted the sentence as suggested, however, we felt it was still important to acknowledge that these remaining schools completed the survey during the aftermath of the pandemic. We have therefore updated the previous sentence, see below.

Pg 7, Ln 161: “All remaining participating schools completed the survey after restrictions had eased and students had returned to in-person schooling, although schools continued to be influenced by the aftermath of the COVID-19 pandemic.”

The sentence: ‘ However, if the broader context of the pandemic schools varied in the methods and extent of contact with students during school closures.’ (P8, 181-2) should be rewritten as: ‘However, in the broader context of the pandemic, schools varied in the methods and extent of contact with students during school closures.’

We have corrected the sentence as suggested (Pg 8, 165).

Attachment Submitted filename: Response to reviewer.docx

# Peer review: Decision Letter 2

**DOI:** 10.1371/journal.pone.0355874.r006

**Authors:** Harikrishnan U

27 Jul 2026

Prevalence of mental health, mental well-being, and school connectedness in UK secondary school students in 2021/2022: results from the South West - School Health Research Network pilot study.

PONE-D-25-55322R2

Dear Dr. Albers,

We’re pleased to inform you that your manuscript has been judged scientifically suitable for publication and will be formally accepted for publication once it meets all outstanding technical requirements.

Within one week, you’ll receive an e-mail detailing the required amendments. When these have been addressed, you’ll receive a formal acceptance letter and your manuscript will be scheduled for publication.

An invoice will be generated when your article is formally accepted. Please note, if your institution has a publishing partnership with PLOS and your article meets the relevant criteria, all or part of your publication costs will be covered. Please make sure your user information is up-to-date by logging into Editorial Manager at Editorial Manager® and clicking the ‘Update My Information' link at the top of the page. For questions related to billing, please contact billing support.

If your institution or institutions have a press office, please notify them about your upcoming paper to help maximize its impact. If they’ll be preparing press materials, please inform our press team as soon as possible -- no later than 48 hours after receiving the formal acceptance. Your manuscript will remain under strict press embargo until 2 pm Eastern Time on the date of publication. For more information, please contact onepress@plos.org.

Kind regards,

Harikrishnan U, Ph.D

Academic Editor

PLOS One

Additional Editor Comments (optional):

Reviewers' comments:

Reviewer's Responses to Questions

**Comments to the Author**

Reviewer #1: All comments have been addressed

Reviewer #3: All comments have been addressed

**********

2. Is the manuscript technically sound, and do the data support the conclusions?

Reviewer #1: Yes

Reviewer #3: (No Response)

**********

3. Has the statistical analysis been performed appropriately and rigorously?

Reviewer #1: Yes

Reviewer #3: (No Response)

**********

4. Have the authors made all data underlying the findings in their manuscript fully available?

The PLOS Data policy

Reviewer #1: Yes

Reviewer #3: (No Response)

**********

5. Is the manuscript presented in an intelligible fashion and written in standard English?

Reviewer #1: Yes

Reviewer #3: (No Response)

**********

Reviewer #1: Manuscript Title Prevalence of mental health, mental well-being, and school connectedness in UK secondary school students in 2021/2022: results from the South West - School Health Research Network pilot study.

Manuscript Number: PONE-D-25-55322R2

Overall Comments: The manuscript is well-written and logically organized. I have no further comments

Reviewer #3: (No Response)

**********

what does this mean?). If published, this will include your full peer review and any attached files.

If you choose “no”, your identity will remain anonymous but your review may still be made public.

**Do you want your identity to be public for this peer review?**  For information about this choice, including consent withdrawal, please see our Privacy Policy

Reviewer #1: No

Reviewer #3: No

**********

Attachment Submitted filename: Reviewers Comments.docx

# Editor report: Acceptance letter

**DOI:** 10.1371/journal.pone.0355874.r007

**Authors:** Harikrishnan U

PONE-D-25-55322R2

PLOS One

Dear Dr. Albers,

I'm pleased to inform you that your manuscript has been deemed suitable for publication in PLOS One. Congratulations! Your manuscript is now being handed over to our production team.

At this stage, our production department will prepare your paper for publication. This includes ensuring the following:

* All references, tables, and figures are properly cited

* All relevant supporting information is included in the manuscript submission,

* There are no issues that prevent the paper from being properly typeset

You will receive further instructions from the production team, including instructions on how to review your proof when it is ready. Please keep in mind that we are working through a large volume of accepted articles, so please give us a few days to review your paper and let you know the next and final steps.

Lastly, if your institution or institutions have a press office, please let them know about your upcoming paper now to help maximize its impact. If they'll be preparing press materials, please inform our press team within the next 48 hours. Your manuscript will remain under strict press embargo until 2 pm Eastern Time on the date of publication. For more information, please contact onepress@plos.org.

You will receive an invoice from PLOS for your publication fee after your manuscript has reached the completed accept phase. If you receive an email requesting payment before acceptance or for any other service, this may be a phishing scheme. Learn how to identify phishing emails and protect your accounts at https://explore.plos.org/phishing.

If we can help with anything else, please email us at customercare@plos.org.

Thank you for submitting your work to PLOS One and supporting open access.

Kind regards,

PLOS ONE Editorial Office Staff

on behalf of

Dr. Harikrishnan U

Academic Editor

PLOS One
